# Identification of robust and reproducible CT‐texture metrics using a customized 3D‐printed texture phantom

**DOI:** 10.1002/acm2.13162

**Published:** 2021-01-12

**Authors:** Bino A. Varghese, Darryl Hwang, Steven Y. Cen, Xiaomeng Lei, Joshua Levy, Bhushan Desai, David J. Goodenough, Vinay A. Duddalwar

**Affiliations:** ^1^ Department of Radiology University of Southern California Los Angeles CA USA; ^2^ The Phantom Laboratory Greenwich NY USA; ^3^ Department of Radiology George Washington University Washington DC USA

**Keywords:** computed tomography, phantom study, radiomics, reproducibility, robustness, texture analysis

## Abstract

**Objective:**

The objective of this study was to evaluate the robustness and reproducibility of computed tomography‐based texture analysis (CTTA) metrics extracted from CT images of a customized texture phantom built for assessing the association of texture metrics to three‐dimensional (3D) printed progressively increasing textural heterogeneity.

**Materials and Methods:**

A custom‐built 3D‐printed texture phantom comprising of six texture patterns was used to evaluate the robustness and reproducibility of a radiomics panel under a variety of routine abdominal imaging protocols. The phantom was scanned on four CT scanners (Philips, Canon, GE, and Siemens) to assess reproducibility. The robustness assessment was conducted by imaging the texture phantom across different CT imaging parameters such as slice thickness, field of view (FOV), tube voltage, and tube current for each scanner. The texture panel comprised of 387 features belonging to 15 subgroups of texture extraction methods (e.g., Gray‐level Co‐occurrence Matrix: GLCM). Twelve unique image settings were tested on all the four scanners (e.g., FOV125). Interclass correlation two‐way mixed with absolute agreement (ICC3) was used to assess the robustness and reproducibility of radiomic features. Linear regression was used to test the association between change in radiomic features and increased texture heterogeneity. Results were summarized in heat maps.

**Results:**

A total of 5612 (23.2%) of 24 090 features showed excellent robustness and reproducibility (ICC ≥ 0.9). Intensity, GLCM 3D, and gray‐level run length matrix (GLRLM) 3D features showed best performance. Among imaging variables, changes in slice thickness affected all metrics more intensely compared to other imaging variables in reducing the ICC3. From the analysis of linear trend effect of the CTTA metrics, the top three metrics with high linear correlations across all scanners and scanning settings were from the GLRLM 2D/3D and discrete cosine transform (DCT) texture family.

**Conclusion:**

The choice of scanner and imaging protocols affect texture metrics. Furthermore, not all CTTA metrics have a linear association with linearly varying texture patterns.

## 1 INTRODUCTION

Texture analysis (TA) can provide quantitative metrics extracted from routine clinical images that can be correlated to and/or predict multiple clinical endpoints.^1^, ^2^, ^3^ Despite having the potential for wide applicability within clinical workflow for tasks such as objective whole lesion assessment and longitudinal disease monitoring, poor standardization of TA, limits its reliability, particularly in multicenter studies.^4^, ^5^, ^6^, ^7^, ^8^

Prior studies have investigated the reliability of computed tomography (CT)‐based TA (CTTA) metrics, by conducting a series of CT imaging experiments using a variety of texture phantoms to evaluate the performance of a CTTA panel on routine imaging protocols.^4^, ^5^, ^9^, ^10^, ^11^, ^12^, ^13^, ^14^, ^15^ The robustness, repeatability, and reproducibility of CTTA metrics are variably sensitive to various scanner and scanning parameters. Studies have reported reliable CTTA metrics, that is, CTTA metrics that are robust, reproducible, and repeatable across different scanners and scanning protocols. The identification of reliable CTTA metrics aid in dimensionality reduction by selecting only reliable metrics and therefore, aid in the development of reliable imaging markers and prediction models.^16^, ^17^ In some studies, the effects of post‐processing techniques that reduce statistical noise while preserving the underlying edges associated with true anatomy or pathology have been explored and shown to bring about significant differences in radiomic reliability compared to when they were not used.^5^, ^18^

Physical phantoms have been used in quantitative imaging to explore and quantify sources of bias and variance, for example, initiatives by the Radiological Society of North America (RSNA), Quantitative Imaging Biomarker Alliance (QIBA),^19^ and the Credence Cartridge Radiomics phantom.^9^ In some cases, virtual phantoms or digital reference objects (DROs) have also been useful for evaluation of software packages that are used to derive quantitative imaging biomarkers. By providing a dataset and a set of metric evaluation that can be accessed by all, radiomics can be rigorously tested in large multi‐institution studies to aid its clinical translation. One such major effort is the Image Biomarker Standardization Initiative (IBSI) that aims to standardize radiomics imaging biomarkers.^20^

While in some cases these calibration objects have been used for standardization of imaging data acquired using diverse scanners, scanning and post processing protocols,^10^, ^11^, ^21^ others use the same approach to identify radiomic metrics that are reliable^5^ (robust, repeatable, and reproducible) so as facilitate big data radiomics using data pooled from these radiomic metrics acquired from multiple institutions.

Although these studies provide some insight into the reliability of CTTA metrics, to the best of our knowledge, a systematic investigation on the association of reliable texture metrics to 3D printed progressively increasing textural heterogeneity across multiple clinical scanners/vendors and imaging protocols has not yet been performed.

In this study, a texture phantom (The Phantom Laboratory, Salem, NY) was designed and constructed explicitly for CTTA metric extraction. Reliability of the extracted CTTA metrics were assessed on four different CT scanners (Philips Brilliance 64, Canon Aquilion Prime 160, GE 16 Lightspeed, and Siemens Sensation 10) and five different imaging variables (slice thickness, field of view, post‐reconstruction filtering, tube voltage, and tube current per scanner). We investigated the association of various CTTA metrics to controlled increases in heterogeneity created by 3D‐printed texture patterns.

## 2 MATERIALS AND METHODS

### 2.A CTTA phantom

**Fig. 1:**
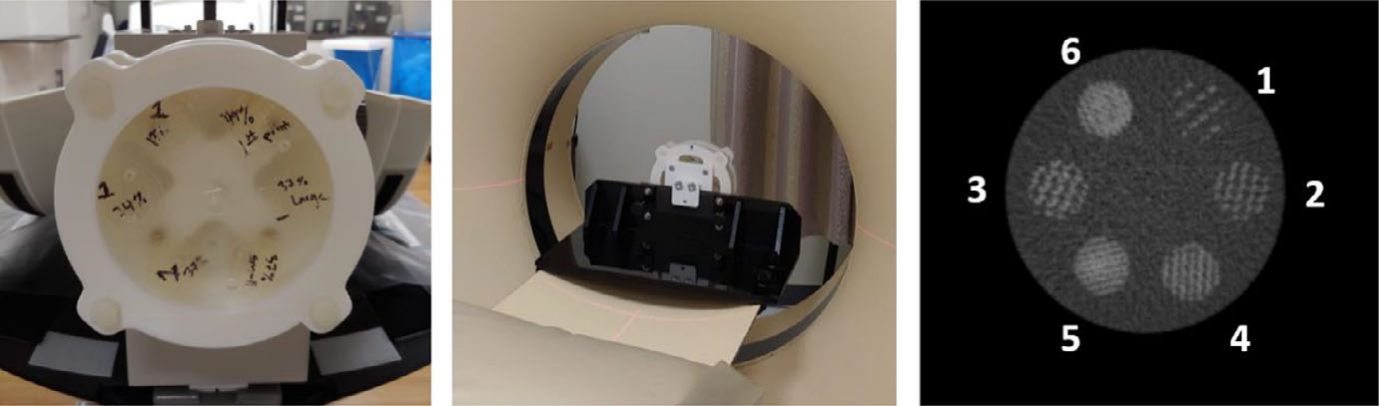
(Left) Texture phantom comprising of six texture patterns. (Middle) Phantom placed within a jig that is designed to securely fasten the phantom to the scanner for image acquisition. (Right) Cross section of texture phantom patterns. (1) through (6) are three‐dimensional‐printed ABS plastic with variable fill levels. The background is a homogenous ABS material. (The window level is −500 HU with a width of 1600 HU).

In this study, we designed and constructed a texture phantom to evaluate the reliability of the CTTA metrics. The phantom comprises of six texture patterns within a homogenous background (Fig. 1). The patterns were 5 cm diameter in a 15‐cm short cylinder. The phantom patterns were made using acrylonitrile butadiene styrene (ABS) first‐order, CT tissue‐equivalent plastic using 3D printing technologies and casting them into tissue density urethane. The patterns 1, 2, 3, and 4 represent texture varying from the smoothest, that is, with a 13% fill, 24% fill, 37% fill, and 46% fill. The basic structural unit of the texture pattern was a letter “J”. The letter “J” was chosen, as it is a simple shape that contains both a straight section and a curve segment and yet, easy to calculate the area and replicate. The spacing between letters J’s in pattern 1 was about 3.5 times larger compared to pattern 4. Rather than increasing heterogeneity by reducing the spacing between features, the heterogeneity can be increased by increasing the size of the structural unit within a fixed area. Patterns 5 and 6 capture this latter variation, wherein at 37% fill, pattern 6 had a smaller size J compared to patterns 1–4, and pattern 6 that had a larger size J compared to patterns 1–4.

The intention was to create a generic phantom that could be imaged using diverse imaging protocols and scanners to identify reliable CTTA metrics and study their association with progressively varying heterogeneity created by 3D‐printed texture patterns (Fig. 1). While a variety of approaches have been used, the current focus has been on creating reproducible geometric patterns, which could be varied in different ways to better understand how changes in patterns drive texture and its analysis. The materials selected for the tests were within the CT tissue density and texture range and provided targeted contrast within our texture patterns. These target Hounsfield number ranges were based on an evaluation of patient images, that is, −500 HU with a width of 1600 HU. It was not our intention to create a lesion‐specific phantom as this would add to the structural complexities involved in creating such a phantom, and introduce variables related to phantom geometry that may affect the performance.

### 2.B CT Imaging

The phantom was scanned on four CT scanners namely, Philips Brilliance 64 (Andover, USA), Canon Aquilion Prime 160 (Tustin, USA), GE 16 Lightspeed (Chicago, USA), and Siemens Sensation 10 (Erlangen, Germany) and four different imaging variables (slice thickness, field of view, tube voltage, and tube current per scanner). Prior to each scan, the phantom was fixed on the CT patient table using a custom‐made jig for the duration of the scan. The image acquisition scanning positioning for each volume was rigidly set to produce identically positioned slices, therefore obviating any need for volume registration. For comparison purposes, in scenarios where the exact match in variable was not available, the closest match was adopted.

### 2.C Volume of interest segmentation

The manual volumes of interest (VOIs) were drawn using image‐rendering software (Synapse 3D, Fujifilm, Stamford CT) across the entire volume of each of the six texture patterns. A few regions of the phantom had air bubbles due to the manufacturing process, and care was taken to exclude these regions when the analysis was performed. Custom MATLAB (Mathworks, Natick, MA, USA) code was used to extract voxel data corresponding to the VOI. Two‐dimensional CTTA was conducted on the orientation that provided the largest diameter in the axial, coronal, or sagittal dimension. Three‐dimensional CTTA was conducted on the whole VOI.

### 2.D Image data

From the six segmented VOIs within the texture phantom, highlighted in Fig. 1, CTTA features were extracted.

### 2.E CTTA extraction

A previously reported CTTA panel, part of the USC radiomics framework, was used to extract the 2D/3D CTTA metrics.^22^, ^23^ The texture panel comprised of 387 features belonging to 15 subgroups of texture extraction methods including intensity, gray‐level co‐occurrence matrix (GLCM) 2D/3D, gray‐level difference matrix (GLDM) 2D/3D, gray‐level run length matrix (GLRLM) 2D/3D, gray‐level size zone matrix (GLSZM) 2D/3D, Law’s Texture Energy (LTE) 2D/3D, neighborhood gray‐tone difference matrix (NGTDM) 2D/3D, fast Fourier transform (FFT) 2D, and discrete cosine transform (DCT) 2D. This radiomics software was chosen, as majority of its CTTA metrics have been benchmarked to IBSI standard values and show <1% variation.^20^ We used a 20‐bin gray‐level quantization. A combination of first‐order statistical measures of texture such as intensity, which accounts for the gray‐level values but not their spatial orientation in an image, second‐order statistical measures of texture such as GLCM, GLDM, GLSZM, GLRLM, and NGTDM that account for both gray intensity and spatial orientation, and higher order statistical metrics of texture such as FFT, DCT, and LTE that provide additional information regarding frequency, assessment at multiple levels (local vs global assessment) was included in the CTTA panel. Mathematical descriptions and definitions of all CTTA metrics have been provided in the supplementary section.

### 2.F Statistical analysis

**Fig. 2:**
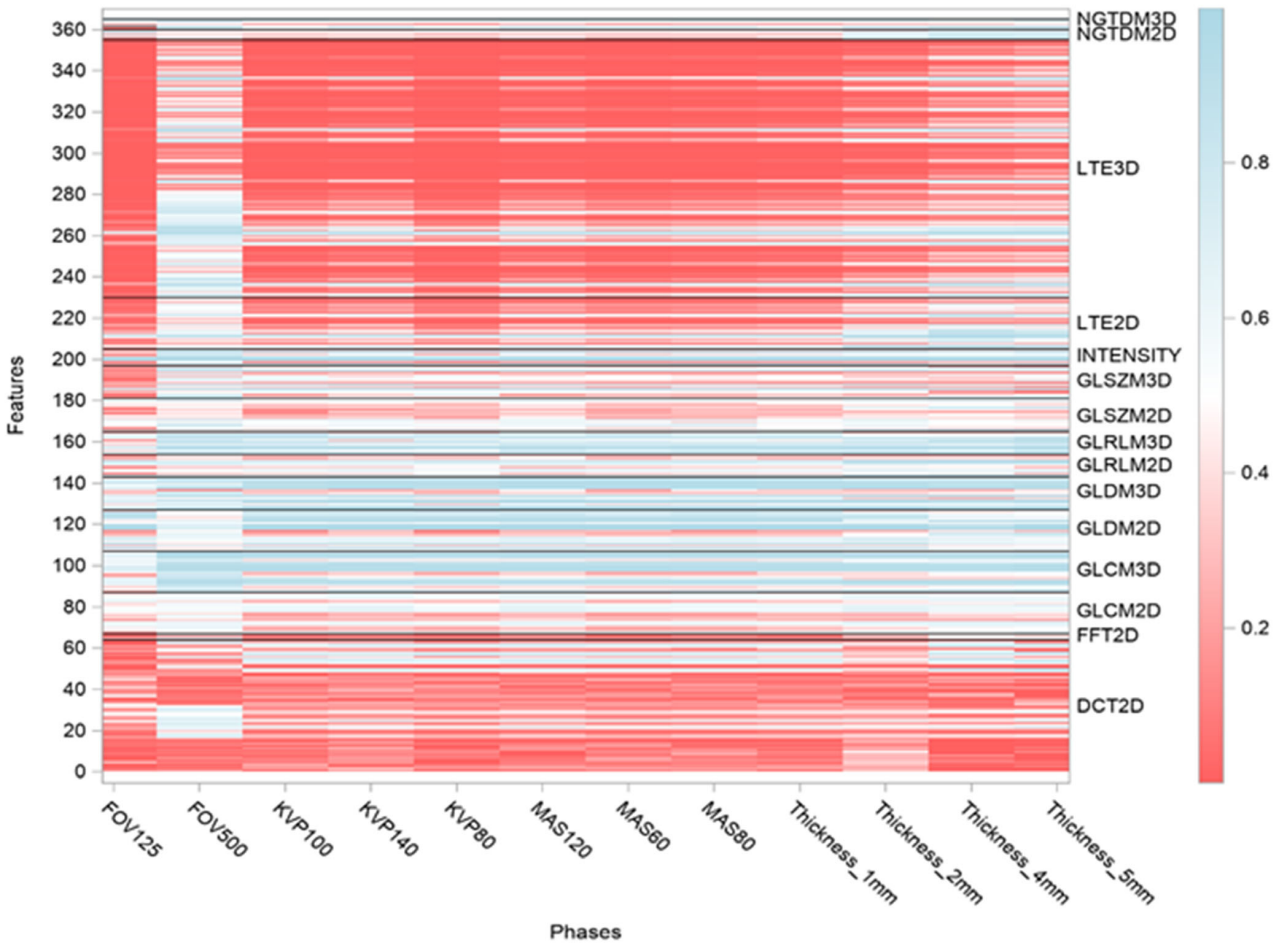
Heat map of radiomic metrics reproducibility, showing the interclass correlation two‐way mixed with absolute agreement (ICC3.1) of each of the radiomic metrics, across the four computed tomography scanners were obtained. Results of the study are presented as a heat map with values ranging from 0 (red) to 1 (blue), that is, poor ICC to high ICC. The texture panel comprised of 365 features belonging to 15 subgroups of texture extraction methods (e.g., GLCM), shown on the y‐axis. Twelve unique image settings (e.g., FOV125) were tested across four scanners, shown on the x‐axis. 22/387 radiomic metrics had same values for all image settings, due to which ICC could not be calculated.

For the reproducibility test, the performance of each radiomic feature under each of the 12 unique image settings was tested across four scanners (x‐axis of Fig. 2). From the images acquired for each setting, six ROIs (ROI 1–6 from Fig. 1) were segmented and analyzed using the texture panel of the USC radiomics framework. The texture panel comprised of 387 features belonging to 15 subgroups of texture extraction methods (y‐axis of Fig. 2). One heat map was used to present the comparison of interclass correlation two‐way mixed with absolute agreement (ICC3) values across the four scanners (Fig. 2). The ICC value ranged from 0(red) to 1 (blue) in the color scale (Fig. 2). In line with literature, features with ICC ≥ 0.90 were considered excellent reproducibility.^24^

Robustness analysis was conducted by pairwise comparisons of 12 settings for each of the four scanners. Therefore, there are 66 × 4 pairs of comparisons in total. Robustness was measured using the interclass correlation two‐way mixed with absolute agreement for single measurement (ICC3.1) of each of the radiomic metrics across the four CT scanners.^25^ The variation in ICC across the different scanners by scanning conditions has been presented as heat maps ranging from 0% (red) to 100% (blue) variation (Figs. S1–S4). In line with literature, features with ICC ≥ 0.90 were considered robust.^24^ For each feature, the 95% confidence interval (CI) of ICC was also produced. If the lower limit of 95% CI for a given ICC was higher than a critical value, for example, 0.8, we can claim the ICC for this feature is statistically significantly higher than the critical value.^34^, ^35^ In this study, we defined the critical value as the following: ≥0.9: identical measurements, ≥0.8: excellent agreement, ≥0.7: good agreement, ≥0.5: fair agreement, <0.5 poor agreement (Figs. S9–S12).

To test the association of the CTTA metrics to the progressively varying texture patterns, a linear regression model was fit from the CTTA metrics acquired from VOIs 1 to 4 and their slopes (β coefficients) were presented as heat maps ranging from −1 (red) to +1 (blue) variation. Red means strong negative linear correlation (decreasing trend), blue means strong positive linear correlation (increasing trend). Linear regression analysis was run on radiomics data pooled from the four scanners. Model integrity was examined using residual plots and distance plots. SAS 9.4 was used for all data analyses.

## 3 RESULTS

Our results indicate the robustness and reproducibility of radiomics metrics are dependent on the scanner and scanning settings. Not all radiomic metrics have a linear association with increased textural heterogeneity.

### 3.A Robustness assessment

Our results indicate that changes in tube voltage (kVp) and exposure (mAs) affect all texture features, but the effect is more on higher order texture metrics such as image transformations: discrete cosine transform (DCT) and Laws transform (LTE) compared to others. With an increase in tube voltage and exposure, the ICC values improved (Fig. 2).

Of all the imaging variables, changes in slice thickness affect all metrics more intensely compared to other imaging variables. As the changes in slice thickness increased, ICC across the metrics increased (Fig. 2).

These observations are consistent across all the four scanners (results in next section)

### 3.B Reproducibility assessment

**Fig. 3:**
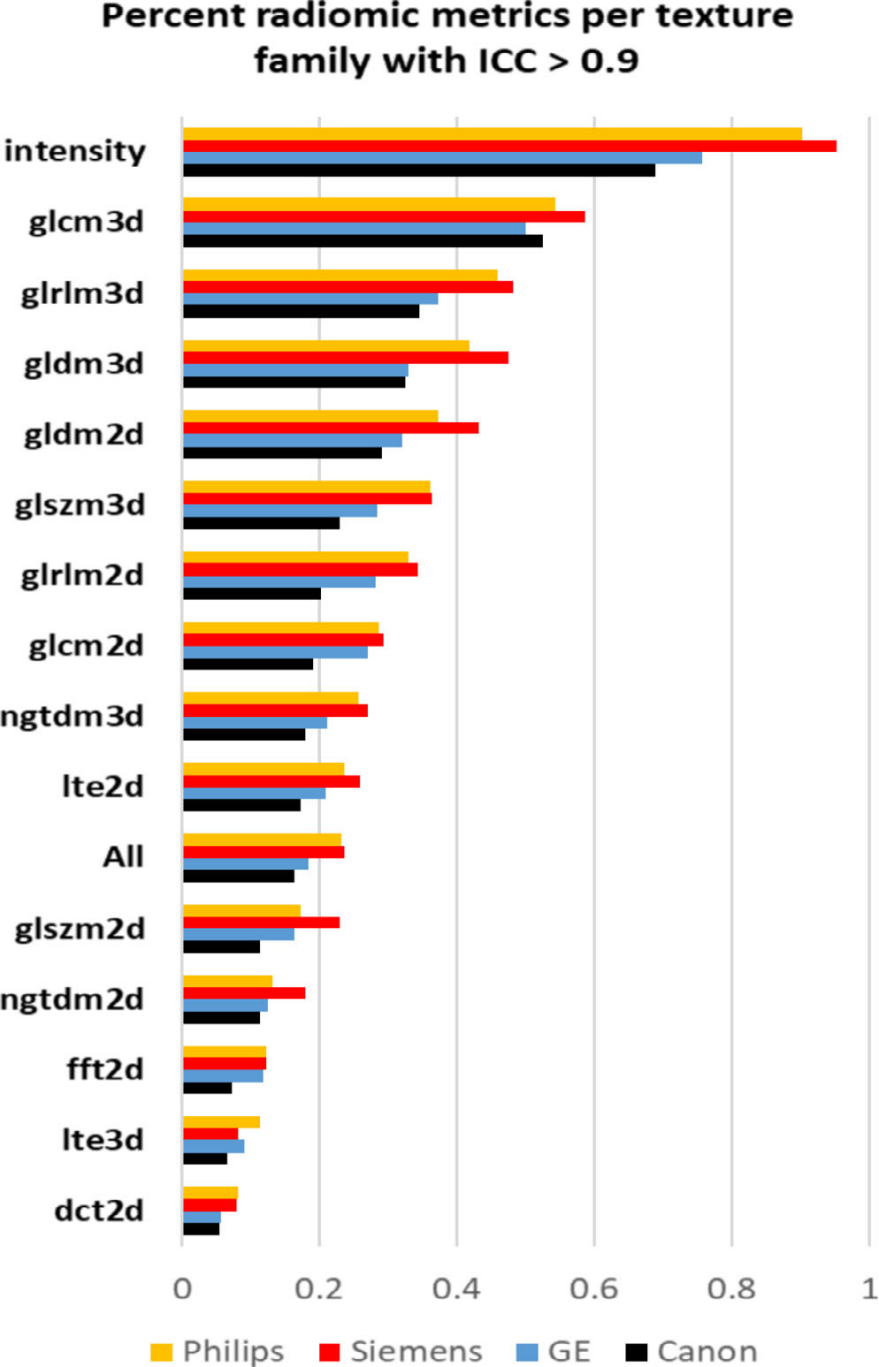
Bar plot showing the percentage of radiomic metrics per texture family (N = 15) that show an ICC ≥ 0.9.

Our results indicate that >40% of intensity, GLCM 3D, and GLRLM 3D features showed ICC ≥ 0.9. This indicates excellent agreement (reproducibility) of these metrics across the four scanners viz Philips Brilliance 64, Canon Aquilion Prime 160, GE 16 Lightspeed, and Siemens Sensation 10 (Fig. 3).

In general, a higher number of metrics with ICC ≥ 0.9 was observed on the Siemens scanner followed by the Philips scanner. The least agreement was observed on the Canon scanner (Fig. 3).

In general, 20% more 3D texture features showed ICC ≥ 0.9 compared to 2D texture metrics (Fig. 3).

### 3.C Assessing the statistical significance of the difference in reliability and robustness based on the analysis of the lower limit of ICC

In general, the results that we obtain for robustness and reproducibility assessment hold true, however, considering the highly stringent acceptance criteria of an alpha of 0.05, fewer metrics per family compared to Figures 2 and 3 showed a high ICC (Figs. S9–S12). Even based on this criteria, intensity, GLCM 3D, and GLRLM 3D showed ICC > 0.8 across maximum number of image settings across the four scanners making them the most reliable texture families.

### 3.D Association with linearly varying textural heterogeneity

**Fig. 4:**
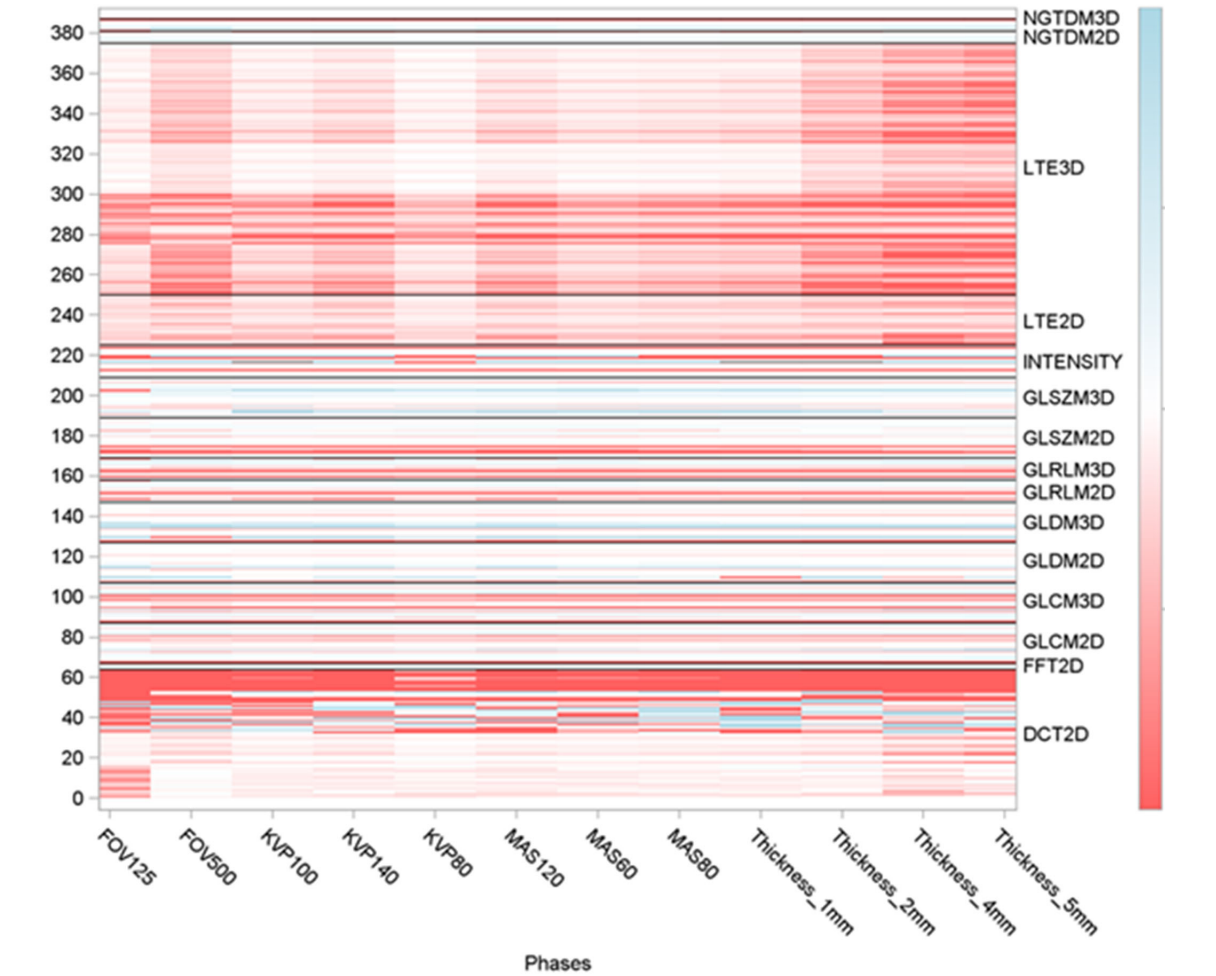
Heat map showing the beta (slope) value of each of the radiomic metrics across the four computed tomography scanners was obtained. Results of the study are presented as a heat map with values ranging from 1 (red) to 1 (blue), that is, negative linear correlation to positive linear correlation. The texture panel comprised of 387 features belonging to 15 subgroups of texture extraction methods (e.g., GLCM), shown on the y‐axis. Twelve unique image settings were tested on all the four scanners (e.g., FOV125), shown on the x axis.

**Fig. 5:**
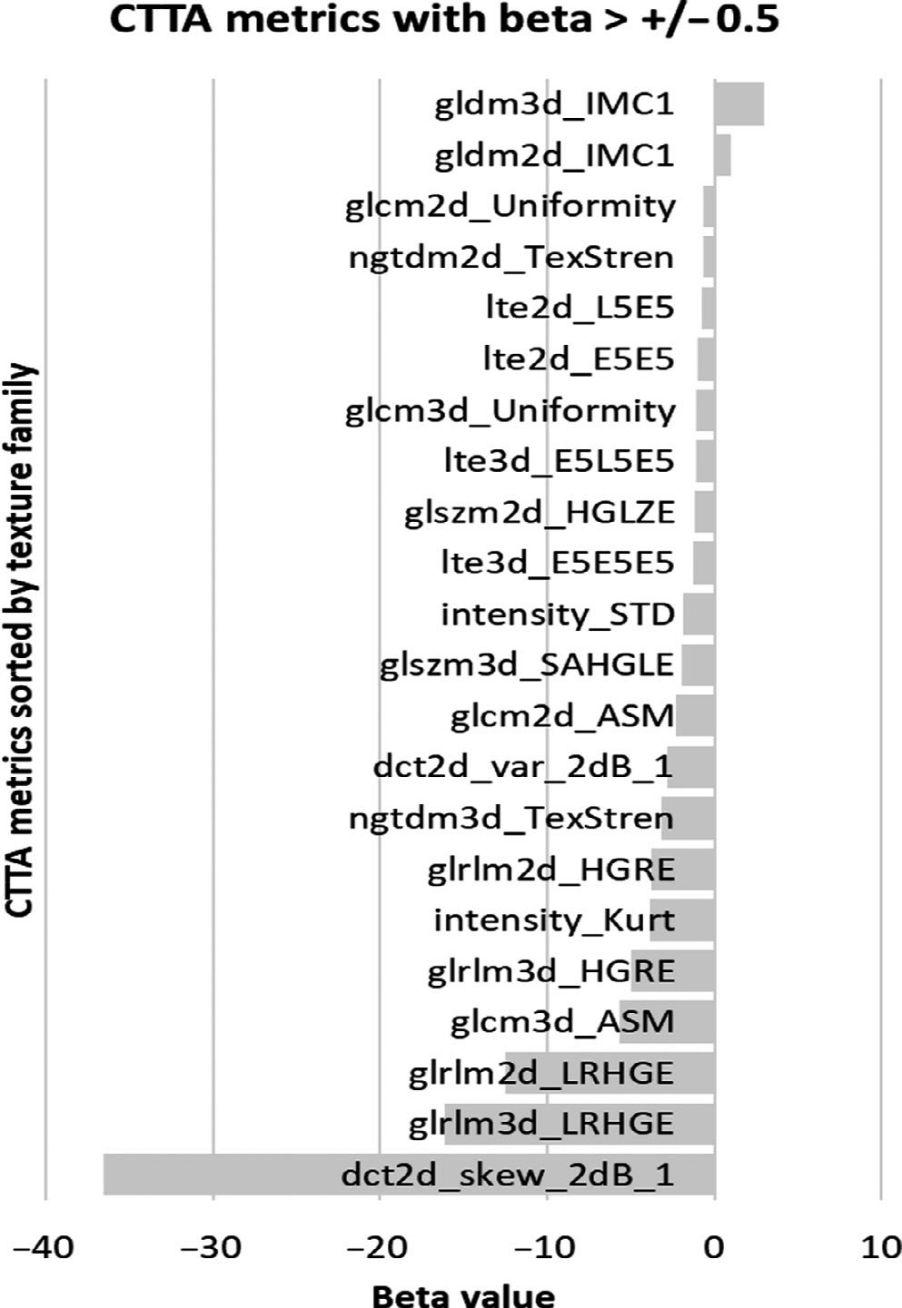
Bar plot showing the beta (slope) values of the radiomic metrics showing a beta value of >0.5 in the positive or negative direction, across the four computed tomography scanners and scanning settings. In case of multiple metrics per family, the top two (highest beta) were included in the plot.

Our results indicate that across all scanner and scanning settings, some texture families were more sensitive to the linear changes in textural patterns than others (Fig. 4), for example, multiple discrete cosine transform (DCT) metrics showed a strong negative association compared to fewer gray‐level difference matrix (GLDM) 3D metrics that showed a strong positive association with the linearly varying textural patterns (Fig. 4). The following observations were made:

1. CTTA metrics: dct2d_skew_2dB_1, glrlm3d_LRHGE, glrlm2d_LRHGE, glcm3d_ASM, glrlm3d_HGRE & intensity_kurtosis showed the five highest beta values. This indicates that these metrics are sensitive to the linear changes in the textural patterns (i.e., ROI 1, 2, 4, and 6) (Fig 5).
2. In general, the only positive association was seen in gldm3d_IMC1, that is, as textural heterogeneity increased from ROI 1 to ROI 6, the value of gldm3d_IMC1 increased linearly. The association was seen in both the 2D and 3D analyses (Fig 5).
3. In general, the radiomic metrics that showed the highest beta values per texture family were the same for 2D and 3D analyses (Fig 5).
4. The beta values were higher in 3D compared to 2D metrics (Fig 5).

Linear trend analysis of radiomic metrics with each of the four scanners has been provided in the Figs. S5–S8.

## 4 DISCUSSION

There is an evolving body of literature addressing the robustness, reproducibility, and repeatability of radiomics‐based texture metrics assessments on a variety of imaging modalities. However, one of the key limitations in the current radiomics reliability studies is that the phantoms used in these studies had not been designed with the specific aim of simulating standard radiomic features.^26^ Therefore, little is known about the association of these reliable radiomic metrics within the context of a controlled variation in radiological texture. Thus, the interpretation of texture metrics, while reliable remains a challenge.

This study systematically investigated the reliability of CTTA metrics derived from routine CT images of a well‐controlled texture phantom setup acquired on four different CT scanners and a combination of four different imaging variables. We report that (a) major differences exist in the number of robust and reproducible features between different scanner/scanning combinations, quantified by the variation in ICC across the different metrics; (b) On an average, a total of 5612 (23.2%) of 24 090 features (i.e., 365 × 66 pair) showed excellent robustness among all imaging variables across all scanners and demonstrated excellent reproducibility (intraclass correlation coefficient ≥ 0.9); (c) Of all the imaging variables, changes in slice thickness affect all metrics more intensely compared to other imaging variables; (d) Changes in kVp and mAs affect mainly higher order texture metrics such as image transformations: discrete cosine transform (DCT) and Laws transform (LTE) compared to others; (e) Select metrics of the DCT, GLCM 3D, GLRLM 2D/3D, and intensity showed high beta values, indicating strong linear associations with linearly varying textural patterns.

Based on the thorough analysis of CTTA robustness and reproducibility, on an average, a total of 5612 (23.2%) of 24 090 features showed excellent robustness across all scanners and demonstrated excellent reproducibility (intraclass correlation coefficient ≥ 0.9). Of these metrics, >40% of intensity, GLCM 3D, and GLRLM 3D features showed an ICC ≥ 0.9 (Fig. 3). In line with these results, other phantom studies on CT also support report these findings. Recent studies by Lu et al., using the Gammex CT ACR 464 phantom and scanning its four water equivalent inserts using routine abdomen protocols on a GE Discovery scanner reported that based on a ranking of the robustness of commonly used CTTA metrics, first‐order texture metrics such as mean, standard deviation, skewness, and kurtosis were more robust than second‐order texture metrics such as GLCM‐energy, correlation, contrast, and homogeneity. While encouraging, the study was performed on only one scanner and using two materials for each pattern. In this study, a similar test using four scanners and six texture patterns reaches the same conclusion. Berenguer et al. using the Credence Cartridge Radiomics phantom for radiomics and restricting the reproducibility analysis of radiomic features using just the polymethyl methacrylate (PMMA) cartridges identified four radiomic features from the intensity group: “60 Percentile,” “Global Median,” “Global Minimum,” and “Kurtosis,” four from the shape group (not assessed here) and two from the GLCM group: “Inverse Difference Normalized” and “Auto Correlation.” In Berenguer et al.’s study, four PMMA cartridges were analyzed P20, P30, P40, and P50. P20 through P50 refer to polylactic acid cartridges with whole percentage from 20% to 50% with respect to solid part. This is very similar to the approach of using ABS with a 13% fill, 24% fill, 37% fill, and 46% fill. The CT window level in the study is −500 HU with a width of 1600 HU. The comparable findings to this study are encouraging. In addition to being reproducible, studies have also shown that first‐order textural features such as intensity metrics are generally more repeatable than high‐order textural features.^5^, ^12^

In this study, image transformation‐based texture methods such as DCT and LTE and texture extraction techniques using gray‐level zoning and gray‐tone differencing showed comparatively poor performance; <12% metrics showed ICC ≥ 0.9. In a study analyzing the stability of 4DCT radiomics metrics in thoracic cancers, Larue et al. also reported higher concordance correlation coefficient values for unfiltered CT images than did the wavelet‐filtered ones.^14^

In general, 20% more 3D texture features showed ICC ≥ 0.9 compared to 2D texture metrics. In agreement to these findings, Zhao et al also found that the 3D features were more reproducible than 2D features across all imaging settings. Fewer lesion pixels in a 2D image likely make the radiomic features more sensitive to changes in imaging variables. In addition, increased spatial resolution along the z‐axis compared to the x/y‐axes provides greater robustness of the 3D features. However, as voxel resolution reduces contributions due to larger partial volume artifacts along the z‐direction may alter this finding.

In this study, the intra‐ and inter‐CT scanner variability of tube voltage and exposure on the reproducibility of the CTTAs was assessed by closely matching the acquisition protocol across for the four scanners analyzed. Automatic scanner/scanning protocols that optimize acquisition were turned off, to gain control over the tube current, tube voltage, and exposure time. Three levels (low, medium, and high) of tube voltage and exposure were predetermined as reported in Table 1. As a higher, tube voltage was used, the percentage of reproducible CTTAs improved from 6.0% (22 of 365) to 8.2% (30 of 365) using ICC > 0.9 as the reference index.^13^ Similarly, the percentage of reproducible CTTAs improved from 6.6% (24 of 365) to 8.2% (30 of 365) when the range of exposure used was increased. Additionally, changes in tube voltage and exposure mainly affected higher order texture metrics such as image transformations: discrete cosine transform (DCT) and Laws transform (LTE) compared to others texture families. The results are supported by similar studies in the literature particularly recent work by Bereguer et al. and Lu et al.^11^, ^13^

**Table 1:** Imaging parameters that were varied across the four scanners.

Imaging variables	Philips brilliance 64 CT	Toshiba aquilion prime 160 CT	GE 16 lightspeed	Siemens sensation 10
Slice thickness (mm)	1, 2, 4, 5	1, 2, 4, 5	1.25, 2.5, 3.75, 5	1, 2, 4, 5
FOV (mm)	125, 500	125, 500	125, 500	125, 500
Tube voltage (kVp)	80, 100, 140	80, 100, 135	80, 100, 140	80, 100, 140
Tube current (effective mAs)	60, 80, 120	60, 80, 120	60, 80, 120	60, 80, 120

It is observed that across all scanners, of all the imaging variables, changes in slice thickness affect all metrics more intensely compared to other imaging variables. Many other studies have reported variation in slice thickness to strongly degraded radiomics reproducibility.^10^, ^15^, ^27^ In general, it is reported that first‐order textural features (intensity) and shape metrics (not considered in this study) are less sensitive to changes in slice thickness compared to higher order textural features. In this study also the magnitude of degradation is observed to be greater for higher order textural features than for intensity. A rationale for this observation may be that a reduction in slice thickness reduces the photon statistics within a slice (unless mAs or kVp is increased; accordingly, which is not the case here), thereby increasing image noise. Therefore, given the variability of pixel spacing and slice thickness in standard of care imaging, it is important to study the impact of these parameters on radiomics features among multiple scanners and multiple vendors in combination with post‐processing and reconstruction kernels. The number of robust and reproducible CTTA metrics increases with increase in slice thickness. This observation is in line with Lu et al.’s radiomics reliability study using the Gammex CT ACR 464 phantom and scanning its four water equivalent inserts using routine abdomen protocols on a GE Discovery scanner.

In this study, across all scanners, for each slice thickness (1–5 mm), the field of view (FOV) was varied from 125 to 500 mm. The axial field of view is one key element that determines the pixel size and hence the spatial sampling in the axial plane, which has an impact on the description of heterogeneity (texture). Consequently, the reduction of pixel size increases spatial resolution (when the other parameters are kept unchanged) but increases image noise as well. In line with this rationale, we observe that across all scanners, a greater number of CTTA metrics show an ICC > 0.9 when the FOV is 500 vs 125 mm.

In this study, “dct2d_skew_2dB_1” which measures the DCT in 2D along the diagonal and reports the skewness, glrlm3d_LRHGE, and glrlm2d_LRHGE which measure the LRGHE in 3D and 2D, respectively, from the GLRLM map of the segmented ROIs, glrlm3d_HGRE which measures the HGRE in 3D from the GLRLM map of the segmented ROIs, glcm3d_ASM which measures the ASM within the GLCM map of the ROI, and the kurtosis measured from the histogram of the ROI were identified to have high beta values. However, the reason for this observation is still being evaluated. For example, it is not clear why transformation techniques such as DCT showed high beta values, compared to other transformation techniques such as fast Fourier transform (FFT) and law’s texture energy (LTE). Also, why texture families such as neighborhood gray tone difference matrix (NGTDM) and GLDM that quantify the difference between a gray value and the average gray value and quantify the absolute value of two pixels with a certain distance is calculated in various directions, respectively, show poor beta values (close to zero). These issues are subject to future research and analysis.

In line with the results from the literature, it is evident from this study that radiomic metrics, particularly those assessing texture are sensitive to changes in scanner and scanning protocols. Therefore, while many studies show diagnostic and prognostic value of CTTA metrics in a variety of different cancers, it must be carefully ensured that these conclusions are based on CTTA metrics that are reproducible and robust to aid generalization across different studies, so as to avoid false positives or negatives.^5^, ^13^ Using this approach, we can identify CTTA features with poor reproducibility (ICC < 0.9) in highly controlled conditions of ROI segmentation, tissue composition, etc. CTTA metrics with poor reproducibility in phantoms are unlikely to be reproducible in multi‐institutional human studies and hence can be removed to alleviate feature dimensionality issues.

The study has several limitations that are common to most investigations of this topic. First, the developed texture phantom is not specific to any particular clinical lesion. Simplified geometries and relatively uniformly dense material compared to actual lesions were adopted to learn about the association of the CTTA metrics with linearly varying texture patterns. In addition, complex texture patterns may not be well captured on phantoms owing to structural instabilities, or limitations of the 3D printing technologies. Therefore, our results may not transfer directly to routine clinical practice, unless the reported phantom‐based feature robustness is reproducible on clinical imaging data from patients.^28^ For example, automated segmentation of phantom ROIs is straightforward considering the clear demarcation of the ROI from the background based on design; however, segmentation can be a serious hurdle in the case on segmenting lesions from patient images.^29^, ^30^ Also, one of the major limitations to understanding radiomic results from a static phantom vs a patient is the influence of motion (involuntary or voluntary) in patients that can profoundly impact scan speed and CT acquisition pattern of sampling.^31^ Yet another issue, to conduct such a study using patient data, is the need for test–retest due to the variation in lesion morphometrics over time. However, it is not feasible to conduct a test–retest experiment using human subjects. Therefore, in this study, a texture phantom is used to assess the reproducibility and robustness of CTTA and understand role of CTTA in multicenter radiomics analysis. Second, only four commonly used CT scanners at our institution and the four most commonly imaging variables were investigated. Post‐processing variables such post reconstruction filters or preprocessing variables such as reconstruction kernels or noise characteristics were not assessed. Published studies have shown these variables to affect radiomic metrics.^11^, ^27^, ^32^ Studies in the literature that assess radiomics reproducibility report concordance correlation coefficient (CCC), interclass correlation coefficient (ICC), or percent change. When reproducibility alone is assessed without repeated measures for a given scanner or modality, the ICC2 (two‐way random ICC) and ICC3 (two‐way mixed ICC) are identical to the concordance correlation coefficient.^2^ However, if reproducibility is assessed with repeated measures, which is equivalent to assessing reproducibility and repeatability at once, only the ICC3 is identical to the concordance correlation coefficient.^33^ In this study, the ICC3 assessment method was chosen and a heat map was used to visualize the results. While there are numerous approaches to extract textural features that can reliably quantify the heterogeneity of the texture patterns, for example, filter (wavelets) and model‐based (fractals) methods, these have not been investigated in our study, as they are not commonly used in open source radiomics pipelines. The difficulty in reaching a consensus on the variables used to implement the technique in a multi‐center setting reduces it widespread applicability. This study is a first step toward trying to understand how these variables affect the radiomic metrics and how they change with linear changes in texture. Future studies exploring more complex texture methods such as fractals and wavelets are warranted. Also, while effects such as noise and motion can affect the radiomics reliability, we did not include it our current study to reduce the complexity of the confounding effects. Using the reliable metrics identified in the current study, future studies can explore the effect of noise and motion on these metrics.

In conclusion, this study shows that CT imaging has the potential to deliver reproducible and robust texture features that may be reliably applied in future clinical studies, particularly those involving multiple centers. Based on these results, it is recommended to use only robust and reproducible features to conduct multicenter radiomics analysis in future studies. More than 40% of intensity, GLCM 3D and GLRLM 3D features showed ICC ≥ 0.9, which makes excellent candidates for creating radiomic signatures with excellent robustness and reproducibility across various CT scanners and scanning protocols. Even based on a stringent significance criteria of alpha of 0.95, intensity, GLCM 3D, and GLRLM 3D showed ICC > 0.8 across maximum number of image settings across the four scanners making them the most reliable texture families. In addition to increasing generalization of results, the identification of these reliable metrics/family of textures helps to narrow down (filter) the rather large number of radiomic features to a reliable few; this alleviates the feature‐dimensionality issue seen in most radiomic studies leading to overfitting of the results and thereby erroneous conclusions. Using the reliable radiomic metrics, data from different studies(published and new) can be pooled to create a relatively large cohort of data for the large‐scale validation of radiomics results, particularly in scenarios where big data principles such as machine‐ or deep‐learning are used to augment classification results.

## AUTHOR CONTRIBUTION

BAV, DJG, and VAD conceived of the presented idea. BAV, DJG, and JL developed the theory and performed the experiments. SYC and XL verified the statistical methods. DH developed the data management pipeline to perform the radiomics analysis and BD provided overall supervision in integrating the activities of the presented idea within the clinical workflow. All authors discussed the results and contributed to the final manuscript.

## CONFLICT OF INTEREST

JL is the president of the Phantom Laboratory, Salem, NY. DG is a consultant to the Phantom Laboratory, Salem, NY

## Supporting information


**Data S1.** A: Mathematical descriptions and definitions of the CT texture metrics. B: Figures of detailed heatmaps showing robustness, reproducibility and linearity of CT texture metrics.
