# Supplementary material for: Identification of robust and reproducible CT‐texture metrics using a customized 3D‐printed texture phantom
Source: J Appl Clin Med Phys. 2021 Jan 12;22(2):98–107. doi: 10.1002/acm2.13162 (PMC7882093; doi:10.1002/acm2.13162)
Supplement: Supplementary file 1 — Data S1. A: Mathematical descriptions and definitions of the CT texture metrics. B: Figures of detailed heatmaps showing robustness, reproducibility and linearity of CT texture metrics. [file ACM2-22-98-s001.pdf]

Supplemental Materials

A. Mathematical descriptions and definitions of the CT texture metrics

Definitions:

- Texture (material): Texture is a measure of the variation of a surface; a rough textured material would have a high rate of change in the high and low points of a surface compared to a smooth textured material.
- Texture (imaging): If on an image of the surface, the global maximum point was assigned a grayscale value of 65536 (for a 16 bit image), the global minimum point was assigned a grayscale value of 0, and all the points in between had an intermediary value between 0 and 65536, scaled by the ratio of its height in comparison to the global maximum point, image of a rough textured material would have a high rate of change in the high and low points of a surface (grayscale value) compared to a smooth textured material.

1. Histogram Analysis/Intensity (3D) (13 metrics): The histogram contains the first-order statistical information about the image (or its fragment). Dividing the values histogram by the total number of pixels in the image one obtains the approximate probability density of occurrence of the intensity (grayscale) levels. Here, eight metrics were used to describe the texture:

a. **Minimum:** Minimum gray value of the pixels forming the region of interest (ROI).

b. **Maximum:** Maximum grayscale value of the pixels forming the ROI.

c. **Mean:** Average grayscale value of the pixels forming the ROI.

d. **Median:** Median grayscale value of the pixels forming the ROI. It is more representative of the distribution, particularly if the distribution is biased.

e. **Variance:** Variance is defined as the expectation of the squared deviation of a random variable from its mean. It is a measure of spread of the sample values from its mean.

f. **Standard deviation (SD):** In statistics, the standard deviation is the usual way of measuring distance from the mean or median (it measures dispersion or variance).

$$SD = \sqrt{\frac{\sum_{i=1}^{M}\sum_{j=1}^{N}(P(i,j) - MEAN)^2}{MN}}$$

where $P(i, j)$ is a grayscale value at a pixel at location row i and column j of an image of size $M \times N$ (row × column).

g. **Quartile Range (QR):** Whereas a range is a measure of where the beginning and end are in a distribution, an interquartile range is a measure of where the middle 50% of the distribution lie.

$$QR = Q_3 - Q_1$$

where Q_3 and Q_1 are the 3rd and 1st quartiles respectively.

h. Skewness (SKEW): Skewness is a measure of the lack of symmetry in a distribution. A symmetric distribution has a skew of zero. A positive skewness indicates a positively skewed distribution and likewise a negative skewness indicates a negatively skewed distribution.

$$SKEW = \frac{\sum_{i=1}^{M} \sum_{j=1}^{N} \left[\frac{(P(i,j) - MEAN}{SD}\right]^3}{MN}$$

i. Kurtosis (KURT): Considered the fourth moment in statistics, kurtosis is indicative of the "peakedness" of the distribution. Technically, it is a measure of how close the values of the distribution are to the mean. A positive kurtosis indicates less outliers and more peaked, likewise a negative kurtosis indicates too many outliers and less peaked.

$$KURT = \frac{\sum_{i=1}^{M} \sum_{j=1}^{N} \left[\frac{(P(i,j) - MEAN)}{SD}\right]^4 - 3}{MN}$$

Histogram analysis is completely based on the distribution of the grayscale values forming the ROI; it provides no information about the spatial relationship of the pixels to each other. Therefore, differentiating 2 completely different texture patterns with the same number but different orientation of black and white pixels is not possible in histogram analysis.

In addition, 4 size-based metrics are calculated: number of voxel dimensions in *x*, *y*, and *z* directions and the volume.

2. Two-dimensional and Three-dimensional Gray Level Co-occurrence Method (GLCM) and Gray Level Difference Method (GLDM) Analysis (20 metrics): These second-order statistical analysis of texture include 2D-GLCM and GLDM analysis and take into account the both pixel intensities and their inter-relationships, thereby providing spatial information of the intensities (2nd order texture analysis) in various forms. For workflow implementation, the number of gray levels were reduced to 12-bit, which was determined to be sufficiently accurate for the study of texture (37). 20 different metrics were calculated: 13 based on the method by Haralick et al. (16) and seven additional metrics.

a. Angular second moment (ASM):

$$ASM = \sum_i \sum_j [P(i,j)]^2$$

ASM reaches its highest value when gray level distribution is constant or repetitive, indicative of a homogenous distribution.

b. Uniformity: Defined as the square root of ASM. Therefore, as in the case of ASM, uniformity reaches its highest value when gray level distribution is constant or repetitive, indicative of a homogenous distribution.

c. Contrast (CON):

$$CON = \sum_i \sum_j P\,(i,j)(i-j)^2$$

Also called sum of squares variance, CON weights pixels in the GLCM/GLDM map exponentially more as their distance from the diagonal increases. A larger value indicates greater variations in gray levels compared to their neighborhood.

d. Dissimilarity (DIS): DIS is similar to contrast, except that the weighting scheme is linear compared to exponential.

e. Homogeneity (HOM):

$$HOM = \sum_{i,j=0}^{N-1} \frac{P_{i,j}}{1 + (i - j)^2}$$

HOM weights pixels in the GLCM/GLDM map exponentially less as their distance from the diagonal increases. A larger value indicates smaller variations in gray levels compared to their neighborhood. This is an inverse metric of CON.

f. Inverse Difference Moment (IDM): IDM is similar to homogeneity, except that the weighting scheme is linear compared to exponential.

$$IDM = \sum_{i,j=0}^{N-1} \frac{P_{i,j}}{1 + (i - j)^2}$$

g. Inverse Difference Moment Normalized (IDMN): IDMN is similar to IDM, except that the weighting scheme is exponential, and it is a measure of the local homogeneity of an image.

$$IDMN = \sum_{i,j=0}^{N-1} \frac{P_{i,j}}{1 + [(i - j)/(N - 1)]^2}$$

h. Entropy (ENT): ENT is a measure of randomness and have a higher value when the distribution is random, as opposed to orderly.

$$ENT = -\sum_{i=1}^{M} \sum_{j=1}^{N} P(i,j) \log P(i,j)$$

i. Correlation (CORR): CORR measures the linear dependency of gray levels on those of neighboring pixels. Technically, it has a value of 0 if uncorrelated, 1 if perfectly correlated.

$$CORR = \sum_{i=1}^{M} \sum_{j=1}^{N} \frac{(i,j)P(i,j) - MEAN_{ROW}MEAN_{COL}}{SD_{ROW}SD_{COL}}$$

j. Information measure of correlation 1 (IMC1): IMC1 is based on the calculations of entropy values of P_x and P_y. For a uniform image (i.e. no pixel changes in that image), the IMC1 is zero. It produces negative values.

$$IMC1 = \frac{-\sum_{i=1}^{M} \sum_{j=1}^{N} P(i,j) \log_2 P(i,j) + \sum_{i=1}^{M} \sum_{j=1}^{N} P(i,j) \log_2 \left[P_x(i), P_y(j)\right]}{MAX\left\{ENT(P_x), ENT\left(P_y\right)\right\}}$$

k. Information measure of correlation 2 (IMC2): For a uniform image (i.e. no pixel changes in that image), the IMC2 is zero. It produces values in the range of 0 to 1.

$$IMC2 = \sqrt{1 - \exp\left[-2\left(\sum_{i=0}^{N-1}\sum_{j=0}^{N-1} P_x(i)P_y(j)\log_2\left[P_x(i), P_y(j)\right] + \sum_{i=0}^{N-1}\sum_{j=0}^{N-1} P_{ij}\log_2 P_{ij}\right)\right]}$$

l. Sum of average (SUMAVG): For an image of single color of no variation, the sum of average values for different angles are 2. Usually, for an image of varied pixel values, the sum of average is high in value.

$$SUMAVG = \sum_{i=0}^{2N-2}(i)P_{x+y}(i) \quad \text{where } P_{x+y}(k) = \sum_{i=0}^{N-1}\sum_{j=0}^{N-1} P(i,j)$$

m. Sum of entropy (SUMENT): As per the definition of entropy, this value goes higher for an image of more variations.

$$SUMENT = \sum_{i=0}^{2N-2} P_{x+y}(i)\log P_{x+y}(i)$$

n. Sum of variance (SUMVAR): Usually for an image of varied pixel values, the sum of variance is high in value.

$$SUMVAR = \sum_{i=0}^{2N-2} P_{x+y}(i)(i - SUMAVER)^2$$

o. Difference of average (DIFAVG) and **difference of entropy (DIFENT)** is calculated similarly to sum of average and entropy respectively, except, differences are calculated instead of summations.

p. Standard deviation (SD) and **mean** are same as those calculated for histogram analysis, except run on GLCM/GLDM maps.

q. Maximum correlation coefficient (MCC):

$$MCC = \sqrt{\text{second largest eigenvalue of } Q} \quad \text{where } Q(i,j) = \sum_k \frac{g(i,k)g(j,k)}{g_x(i)g_y(k)}$$

r. Maximum probability: This metric captures the occurrences of the most dominant pair of neighboring intensity values.

s. Root mean square (RMS): RMS computes the root mean square value of each row or column of the input, along vectors of a specified dimension of the input, or of the entire input.

$$RMS = \sqrt{\frac{\sum_{i=1}^{M} MEAN(i,j)^2}{M}}$$

3. Fast Fourier Transform (FFT) Analysis (3 metrics): Specifically, a 512-point FFT was applied to all tumor images. Using the built-in MATLAB implementation of the FFT algorithm (FFT2), we extracted the individual frequencies, amplitude (how much frequency of a given type is present), and phase (where in the image the frequency is present) of the original image. The resultant magnitude and phase of the FFT across all images were analyzed. Three metrics were defined. In all cases, the harmonics analysis was limited between 15% and 95% of maximum spatial frequency within the tumor. These cutoffs were chosen to avoid inclusion of

low-frequency content (i.e. tumor size effect and noise) and high-frequency noise. The selected band-pass frequencies correspond to the spatial frequencies within the tumor.

a. Entropy of FFT magnitude (E_FFT_Mag): Diversity (Randomness) measure in the magnitude of FFT harmonics.

$$E\_FFT\_Mag = -\sum_{k=0}^{n} P_k \log_2 P_k$$

where P_k is each harmonic from the FFT transformed (magnitude) tumor image.

The E_FFT_Mag of a homogenous texture should be smaller compared to that of a heterogeneous texture.

b. Entropy of FFT phase (E_FFT_Phase): Diversity (Randomness) measure in the phase of FFT harmonics.

$$E\_FFT\_Phase = -\sum_{k=0}^{n} P_k \log_2 P_k$$

where P_k is every harmonic from the FFT transformed (phase) tumor image.

The E_FFT_Phase of a homogenous texture should be smaller compared to that of a heterogeneous texture.

c. Complexity index (CI): Sum of the amplitude of all FFT harmonics

$$CI = \sum_{k=0}^{n} P_k$$

where P_k is every harmonic from the FFT transformed (amplitude) tumor image.

The CI of a homogenous texture should be a smaller value compared to the CI of a heterogeneous texture.

4. Two-dimensional and Three-dimensional Gray Level Run-Length Matrix (GLRLM) (11 metrics):

a. Gray Level Non-Uniformity (GLN) measures the distribution of runs over the gray values (39). A lower value indicates higher similarity in intensity values.

$$GLN = \frac{\sum_{i=1}^{N_g}\left[\sum_{j=1}^{N_r} P(i,j)\right]^2}{N_z}$$

b. High Gray Level Run Emphasis (HGLRE) measures the gray-level analogue to long runs emphasis (i.e. high gray levels).

$$HGLRE = \frac{\sum_{i=1}^{N_g}\sum_{j=1}^{N_r} P(i,j)i^2}{N_z}$$

c. Long Run Emphasis (LRE) measures the distribution of long runs. Coarser textures have higher LRE value.

$$LRE = \frac{\sum_{i=1}^{N_g}\sum_{j=1}^{N_r} P(i,j)j^2}{N_z}$$

d. Low Gray Level Run Emphasis (LGLRE)

$$LGLRE = \frac{\sum_{i=1}^{N_g}\sum_{j=1}^{N_r} \frac{P(i,j)}{i^2}}{N_z}$$

e. Long Run Low Gray Level Emphasis (LRLGLE) measures the runs in the upper right quadrant of the GLRLM. This is where long run lengths and low gray levels are located (40).

$$LRLGLE = \frac{\sum_{i=1}^{N_g}\sum_{j=1}^{N_r} \frac{P(i,j)j^2}{i^2}}{N_z}$$

f. Long Run High Gray Level Emphasis (LRHGLE) measures the runs in the lower right quadrant of the GLRLM. This is where long run lengths and high gray levels are located (40).

$$LRHGLE = \frac{\sum_{i=1}^{N_g}\sum_{j=1}^{N_r} P(i,j)i^2j^2}{N_z}$$

g. Run Length Non-Uniformity (RLN) measures the distribution of runs over the run lengths (Galloway, 1975). Runs that are equally distributed along run lengths show small RLN.

$$RLN = \frac{\sum_{i=1}^{N_r}\left[\sum_{j=1}^{N_g} P(i,j)\right]^2}{N_z}$$

h. Run Percentage (RP) measures the ratio of the number of realized runs to the maximum number of potential runs. Strongly linear or highly uniform ROI volumes feature a low RP.

$$RP = \frac{N_z}{N_p}$$

i. Short Run Low Gray Level Emphasis (SRLGLE) measures the runs in the upper left quadrant of the GLRLM. This is where short run lengths and low gray levels are located (40).

$$SRLGLE = \frac{\sum_{i=1}^{N_g}\sum_{j=1}^{N_r} \frac{P(i,j)}{i^2j^2}}{N_z}$$

j. Short Run High Gray Level Emphasis (SRHGLE) measures the runs in the lower left quadrant of the GLRLM. This is where short run lengths and high gray levels are located (40).

$$SRHGLE = \frac{\sum_{i=1}^{N_g}\sum_{j=1}^{N_r} \frac{P(i,j)i^2}{j^2}}{N_z}$$

k. Short Run Emphasis (SRE) measures the distribution of short runs. Fine textures show higher SRE values.

$$SRE = \frac{\sum_{i=1}^{N_g}\sum_{j=1}^{N_r} \frac{P(i,j)}{j^2}}{N_z}$$

5. Two-dimensional and Three-dimensional Gray Level Size-Zone Matrix (GLSZM) (20 metrics):

a. **Small Area Emphasis (SAE)** measures small zones. Fine textures show a higher SAE value.

$$SAE = \frac{\sum_{i=1}^{N_g} \sum_{j=1}^{N_s} \frac{P(i,j)}{j^2}}{N_z}$$

b. **Large Area Emphasis (LAE)** measures large zones. Fine textures show a lower LAE value.

$$LAE = \frac{\sum_{i=1}^{N_g} \sum_{j=1}^{N_s} P(i,j) j^2}{N_z}$$

c. **Gray Level Non-Uniformity (GLN)** measures the distribution of zone counts over the gray values. Equally distributed zone counts along gray levels show low GLN.

$$GLN = \frac{\sum_{i=1}^{N_g} \left[\sum_{j=1}^{N_s} P(i,j)\right]^2}{N_z}$$

d. **Gray Level Non-Uniformity Normalized (GLNN)** is a normalized version of the GLN.

$$GLNN = \frac{\sum_{i=1}^{N_g} \left[\sum_{j=1}^{N_s} P(i,j)\right]^2}{{N_z}^2}$$

e. **Size Zone Non-Uniformity (SZN)** measures the distribution of zone counts over various zone sizes. Equally distributed zone counts along zone sizes are associated with low SZN.

$$SZN = \frac{\sum_{j=1}^{N_s} \left[\sum_{i=1}^{N_g} P(i,j)\right]^2}{N_z}$$

f. **Size Zone Non-Uniformity Normalized (SZNN)** is a normalized version of SZN.

$$SZNN = \frac{\sum_{j=1}^{N_s} \left[\sum_{i=1}^{N_g} P(i,j)\right]^2}{{N_z}^2}$$

g. **Zone Percentage (ZP)** measures the ratio of the number of realized zones to the maximum number of potential zones. Highly uniform ROIs produce a low ZP.

$$ZP = \frac{N_z}{N_p}$$

h. **Low Gray Level Zone Emphasis (LGLZE)** is a measure of gray-level analogue to small zone emphasis. Here, low gray levels are measured, instead of small zone sizes.

$$LGLZE = \frac{\sum_{i=1}^{N_g} \sum_{j=1}^{N_s} \frac{P(i,j)}{i^2}}{N_z}$$

i. **High Gray Level Zone Emphasis (HGLZE)** is a measure of gray-level analogue to large zone emphasis. The feature emphasizes high gray levels.

$$HGLZE = \frac{\sum_{i=1}^{N_g}\sum_{j=1}^{N_s} P(i,j) i^2}{N_z}$$

j. **Small Area Low Gray Level Emphasis (SALGLE)** measures zone counts within the upper left quadrant of the GLSZM. This is where small zone sizes and low gray levels are located.

$$SALGLE = \frac{\sum_{i=1}^{N_g}\sum_{j=1}^{N_s} \frac{P(i,j)}{i^2 j^2}}{N_z}$$

k. **Small Area High Gray Level Emphasis (SAHGLE)** measures zone counts in the lower left quadrant of the GLSZM. This is where small zone sizes and high gray levels are located.

$$SAHGLE = \frac{\sum_{i=1}^{N_g}\sum_{j=1}^{N_s} \frac{P(i,j) i^2}{j^2}}{N_z}$$

l. **Large Area Low Gray Level Emphasis (LALGLE)** measures zone counts in the upper right quadrant of the GLSZM. This is where large zone sizes and low gray levels are located.

$$LALGLE = \frac{\sum_{i=1}^{N_g}\sum_{j=1}^{N_s} \frac{P(i,j) j^2}{i^2}}{N_z}$$

m. **Large Area High Gray Level Emphasis (LAHGLE)** measures zone counts in the lower right quadrant of the GLSZM. This is where large zone sizes and high gray levels are located.

$$LAHGLE = \frac{\sum_{i=1}^{N_g}\sum_{j=1}^{N_s} P(i,j) i^2 j^2}{N_z}$$

n. **Gray Level Variance** measures the variance in zone counts for the gray levels.

$$\sum_{i=1}^{N_g}\sum_{j=1}^{N_s} p(i,j)(i-\mu)^2 \quad \text{where } \mu = \sum_{i=1}^{N_g}\sum_{j=1}^{N_s} p(i,j) i$$

o. **Size Zone Variance** measures the variance in zone counts across different zone sizes.

$$\sum_{i=1}^{N_g}\sum_{j=1}^{N_s} p(i,j)(j-\mu)^2 \quad \text{where } \mu = \sum_{i=1}^{N_g}\sum_{j=1}^{N_s} p(i,j) j$$

p. N_g**:** Total number of gray levels in the image.

q. N_s**:** Total number of zone sizes in the image.

r. N_p**:** Total number of voxels in the image.

s. N_z**:** Number of zones in the image.

t. μ**:** Mean

$$\mu = \sum_{i=1}^{N_g}\sum_{j=1}^{N_s} p(i,j) i$$

6. Laws' texture energy analysis: Laws developed a texture-energy approach that measures the amount of variation within a fixed size window (41). A set of 5 × 5 convolution masks are used to compute texture energy. The masks are defined as follows:

a. **L5** (**L**evel) = $[1 \quad 4 \quad 6 \quad 4 \quad 1]$

b. **E5** (**E**dge) = $[-1 \quad -2 \quad 0 \quad 2 \quad 1]$

c. **S5** (**S**pot) = $[-1 \quad 0 \quad 2 \quad 0 \quad -1]$

d. **R5** (**R**ipple) = $[1 \quad -4 \quad 6 \quad -4 \quad 1]$

e. **W5** (**W**ave) = $[-1 \quad 2 \quad 0 \quad -2 \quad 1]$

25 unique combinations for 2D analysis (e.g. L5E5, L5L5, L5S5, L5R5, L5W5, etc.). 109 unique combinations for 3D analysis (e.g. L5E5S5, L5E5R5, L5E5W5, etc.).

7. Neighboring Gray Tone Difference Matrix (NGTDM) (6 metrics): An NGTDM quantifies the difference between a gray value and the average gray value of its neighbors within distance δ.

a. Coarseness:

$$\frac{1}{\sum_{i=1}^{N_g} p_i s_i}$$

b. Contrast:

$$\left(\frac{1}{N_{g,p}(N_{g,p}-1)}\sum_{i=1}^{N_g}\sum_{j=1}^{N_g} p_i p_j (i-j)^2\right)\left(\frac{1}{N_{v,p}}\sum_{i=1}^{N_g} s_i\right) \quad \text{where } p_i \neq 0, p_j \neq 0$$

c. Busyness:

$$\frac{\sum_{i=1}^{N_g} p_i s_i}{\sum_{i=1}^{N_g}\sum_{j=1}^{N_g} |ip_i - jp_j|} \quad \text{where } p_i \neq 0, p_j \neq 0$$

d. Complexity:

$$\frac{1}{N_{v,p}}\sum_{i=1}^{N_g}\sum_{j=1}^{N_g} |i-j| \frac{p_i s_i + p_j s_j}{p_i + p_j} \quad \text{where } p_i \neq 0, p_j \neq 0$$

e. Strength:

$$\frac{\sum_{i=1}^{N_g}\sum_{j=1}^{N_g} (p_i + p_j)(i-j)^2}{\sum_{i=1}^{N_g} s_i} \quad \text{where } p_i \neq 0, p_j \neq 0$$

8. Discrete Cosine Transform (DCT) (64 metrics): DCT decomposes an image into discrete cosine waves at various resolutions (levels). Wavelets in general provide good frequency resolution for low frequency and good temporal resolution for high frequency. Here, we perform 2D analysis.

a. **4 levels:** 1, 2, 3, and 4

b. **4 directions:** vertical (v), horizontal (h), diagonal1(d1), and diagonal2 (d2)

c. **4 metrics:** kurtosis, mean, skewness, and variance (see section on histogram analysis)

B. Figures

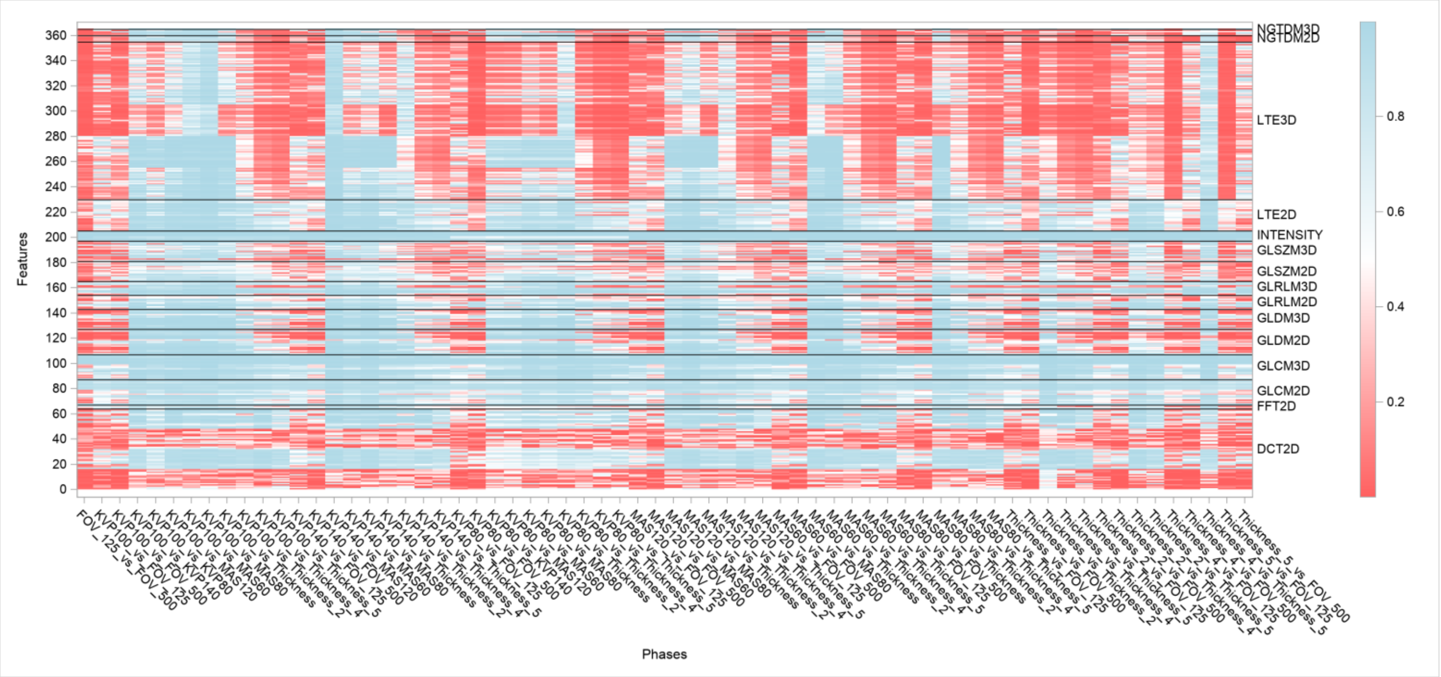

Figure 1: *Heatmap of radiomic metrics robustness, showing the interclass correlation 2-way mixed with absolute agreement (ICC3.1) of each of the radiomic metrics, within the* ***Siemens Sensation 10*** *scanner. Results of the study are presented as a heatmap with values ranging from 0 (red) to 1 (blue) i.e., poor ICC to high ICC. The texture panel comprised of 365 features belonging to 15 subgroups of texture extraction methods (e.g. GLCM), shown on the y-axis. The heatmap shows 12 unique image settings (e.g. FOV125), for comparisons across scanners.*

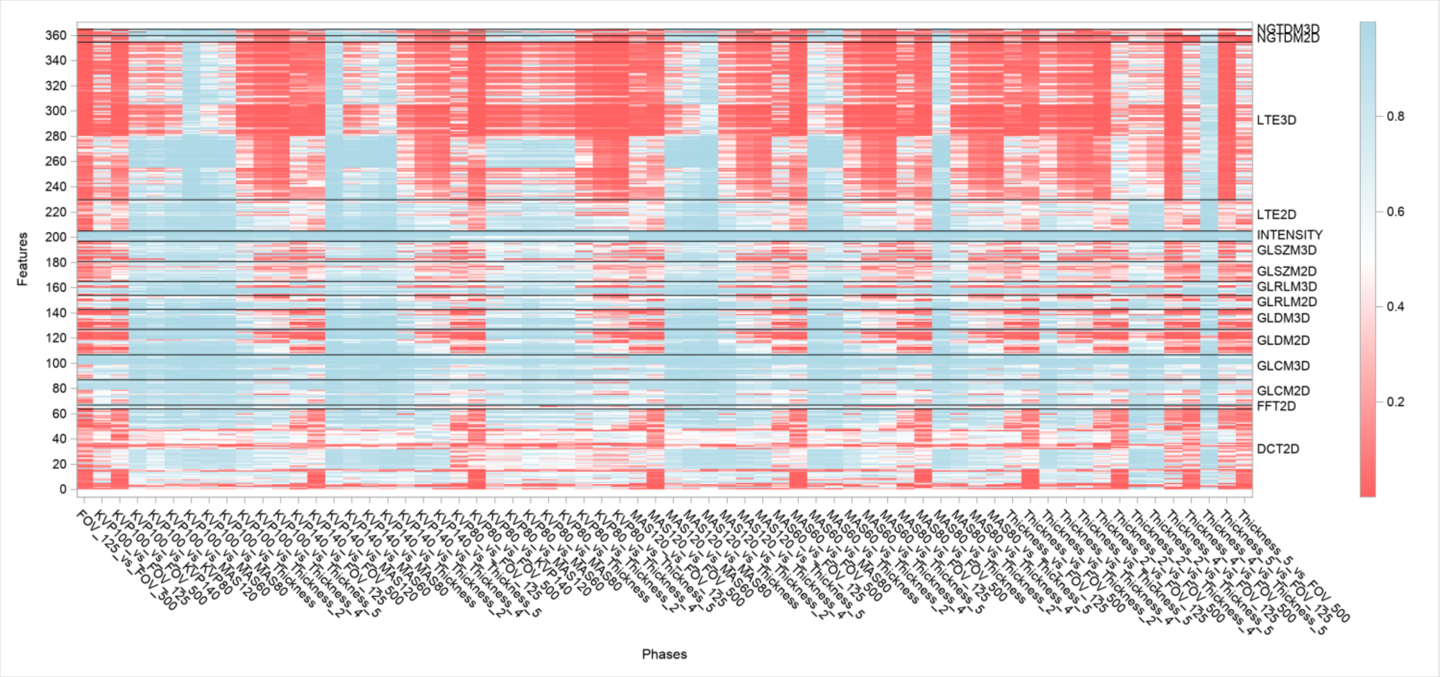

Figure 2: *Heatmap of radiomic metrics robustness, showing the interclass correlation 2-way mixed with absolute agreement (ICC3.1) of each of the radiomic metrics, within the* **Philips Brilliance 64** *scanner. Results of the study are presented as a heatmap with values ranging from 0 (red) to 1 (blue) i.e., poor ICC to high ICC. The texture panel comprised of 365 features belonging to 15 subgroups of texture extraction methods (e.g. GLCM), shown on the y-axis. The heatmap shows 12 unique image settings (e.g. FOV125), for comparisons across scanners.*

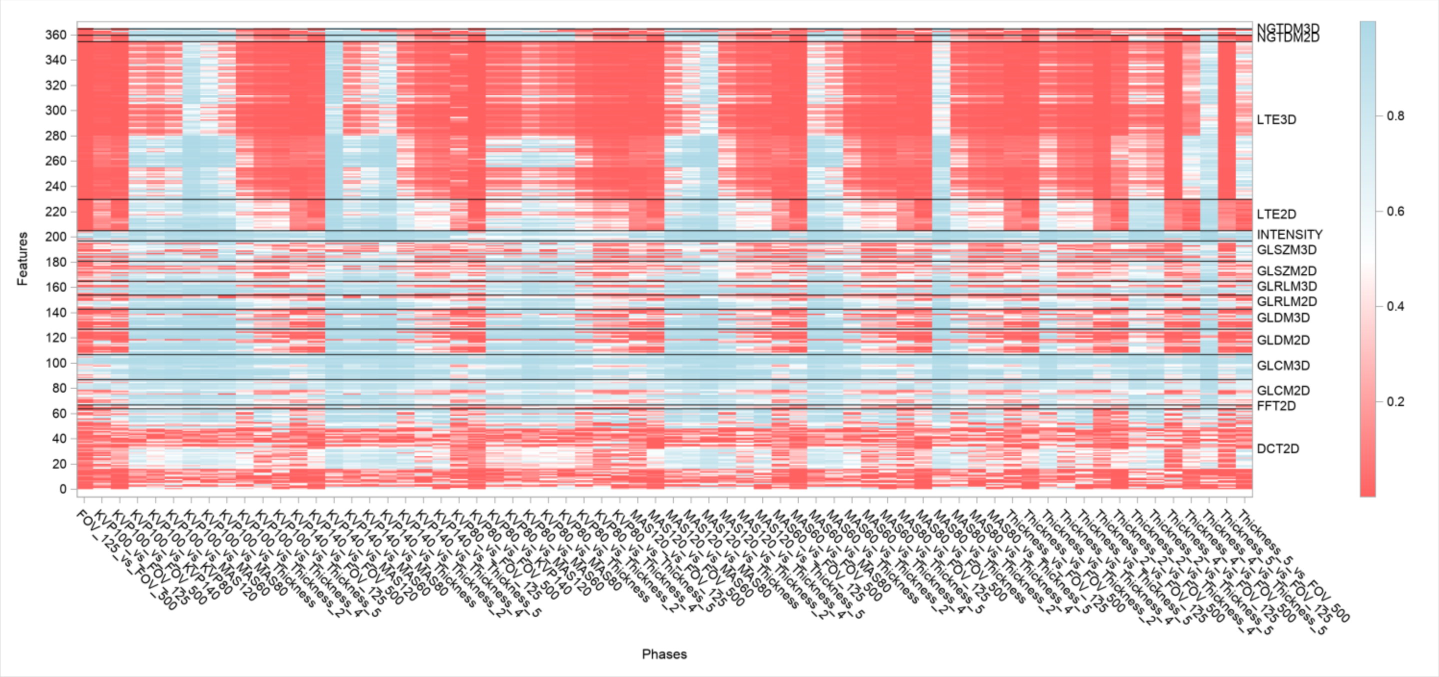

Figure 3: *Heatmap of radiomic metrics robustness, showing the interclass correlation 2-way mixed with absolute agreement (ICC3.1) of each of the radiomic metrics, within the* **Canon Aquilion Prime 160** *scanner. Results of the study are presented as a heatmap with values ranging from 0 (red) to 1 (blue) i.e., poor ICC to high ICC. The texture panel comprised of 365 features belonging to 15 subgroups of texture extraction methods (e.g. GLCM), shown on the y-axis. The heatmap shows 12 unique image settings (e.g. FOV125), for comparisons across scanners.*

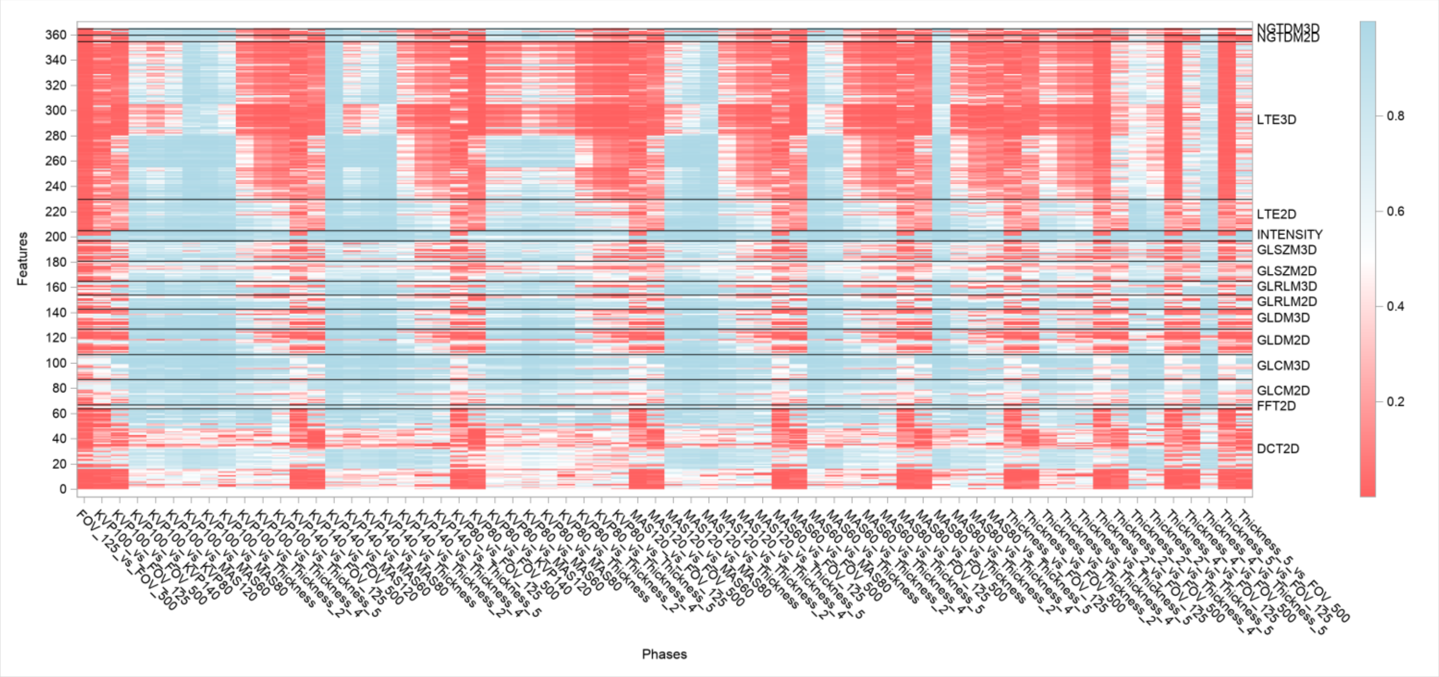

Figure 4: *Heatmap of radiomic metrics robustness, showing the interclass correlation 2-way mixed with absolute agreement (ICC3.1) of each of the radiomic metrics, within the* ***GE 16 Lightspeed*** *scanner. Results of the study are presented as a heatmap with values ranging from 0 (red) to 1 (blue) i.e., poor ICC to high ICC. The texture panel comprised of 365 features belonging to 15 subgroups of texture extraction methods (e.g. GLCM), shown on the y-axis. The heatmap shows 12 unique image settings (e.g. FOV125), for comparisons across scanners.*

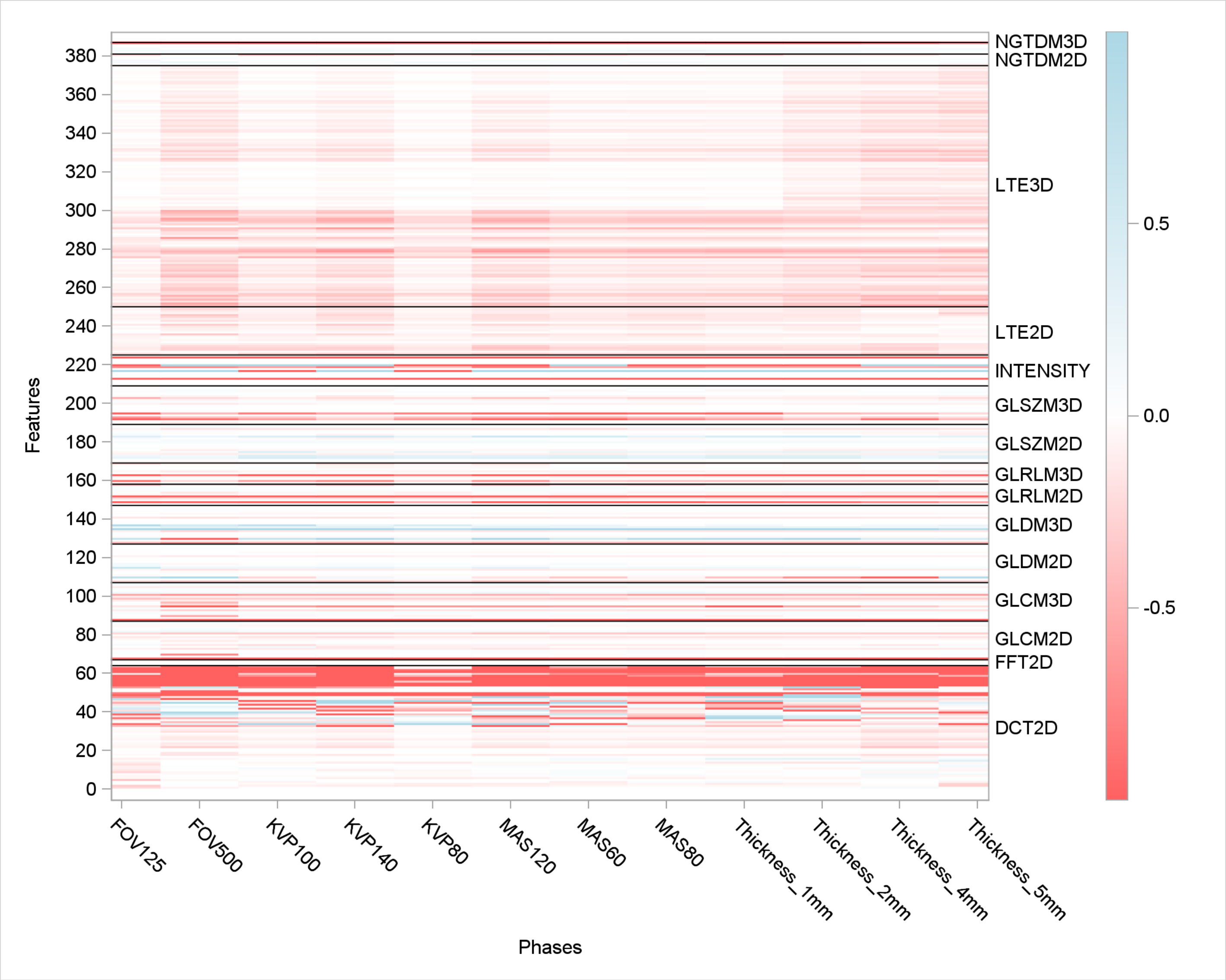

Figure 5: *Heatmap showing the beta (slope) value of each of the radiomic metrics within the* ***Siemens Sensation 10*** *scanner. Results of the study are presented a heatmap with values ranging from -1 (red) to 1 (blue) i.e., negative linear correlation to positive linear correlation. The texture panel comprised of 387 features belonging to 15 subgroups of texture extraction methods (e.g. GLCM), shown on the y-axis. 12 unique image settings were tested (e.g. FOV125), shown on the x-axis.*

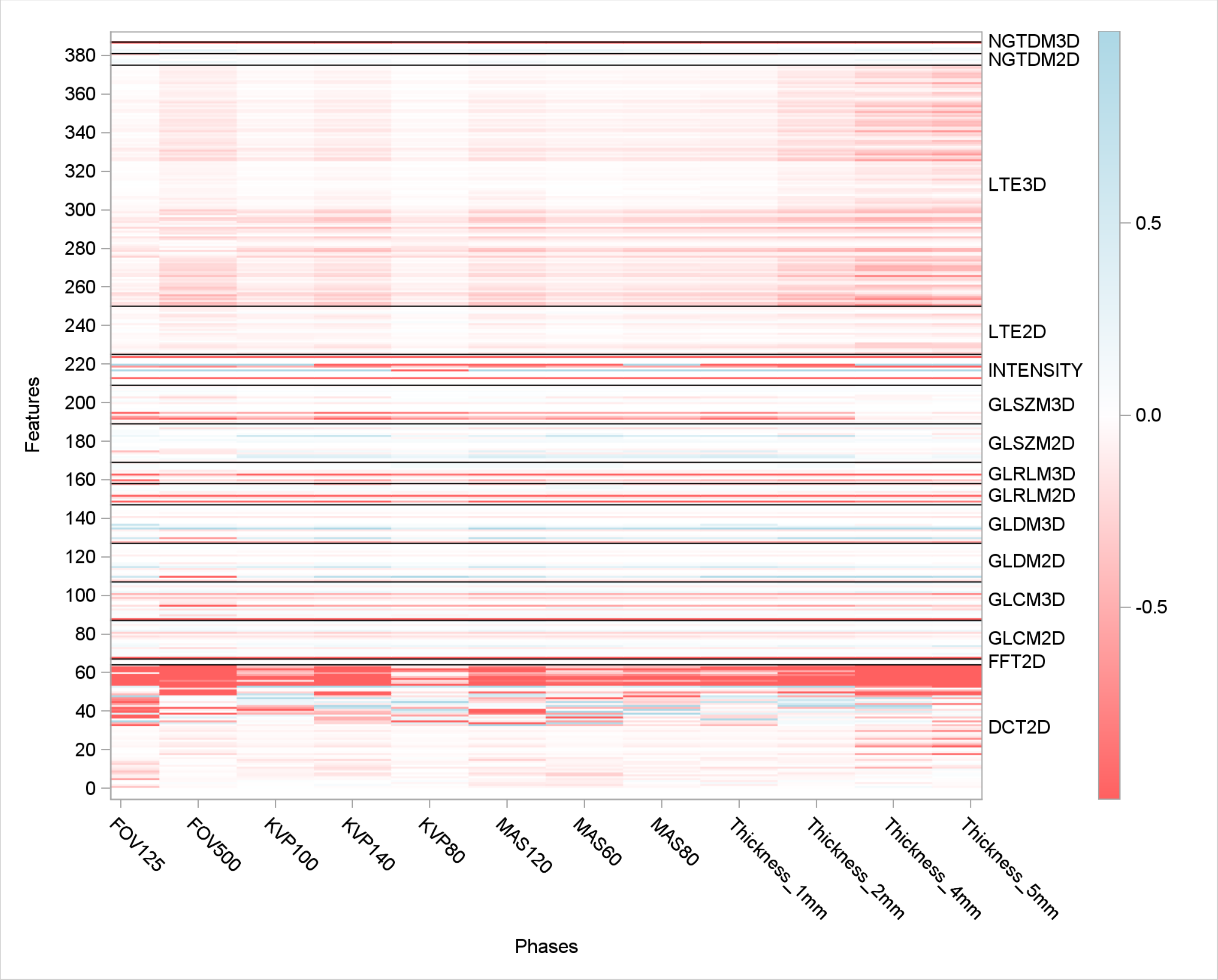

Figure 6: *Heatmap showing the beta (slope) value of each of the radiomic metrics within the* ***Philips Brilliance 64*** *scanner. Results of the study are presented a heatmap with values ranging from -1 (red) to 1 (blue) i.e., negative linear correlation to positive linear correlation. The texture panel comprised of 387 features belonging to 15 subgroups of texture extraction methods (e.g. GLCM), shown on the y-axis. 12 unique image settings were tested (e.g. FOV125), shown on the x-axis.*

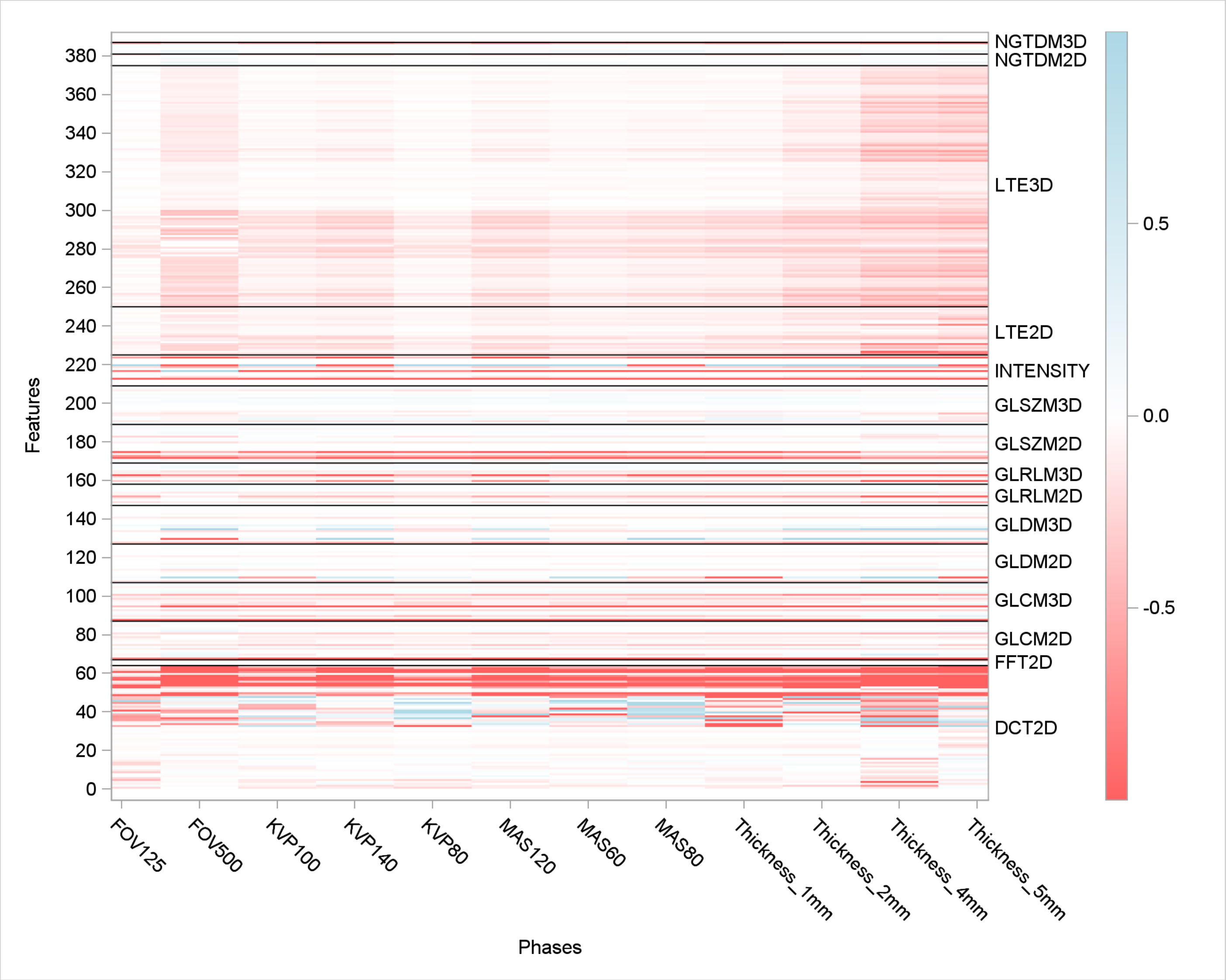

***Figure** 7: Heatmap showing the beta (slope) value of each of the radiomic metrics within the **Canon Aquilion Prime 160** scanner. Results of the study are presented a heatmap with values ranging from -1 (red) to 1 (blue) i.e., negative linear correlation to positive linear correlation. The texture panel comprised of 387 features belonging to 15 subgroups of texture extraction methods (e.g. GLCM), shown on the y-axis. 12 unique image settings were tested (e.g. FOV125), shown on the x-axis.*

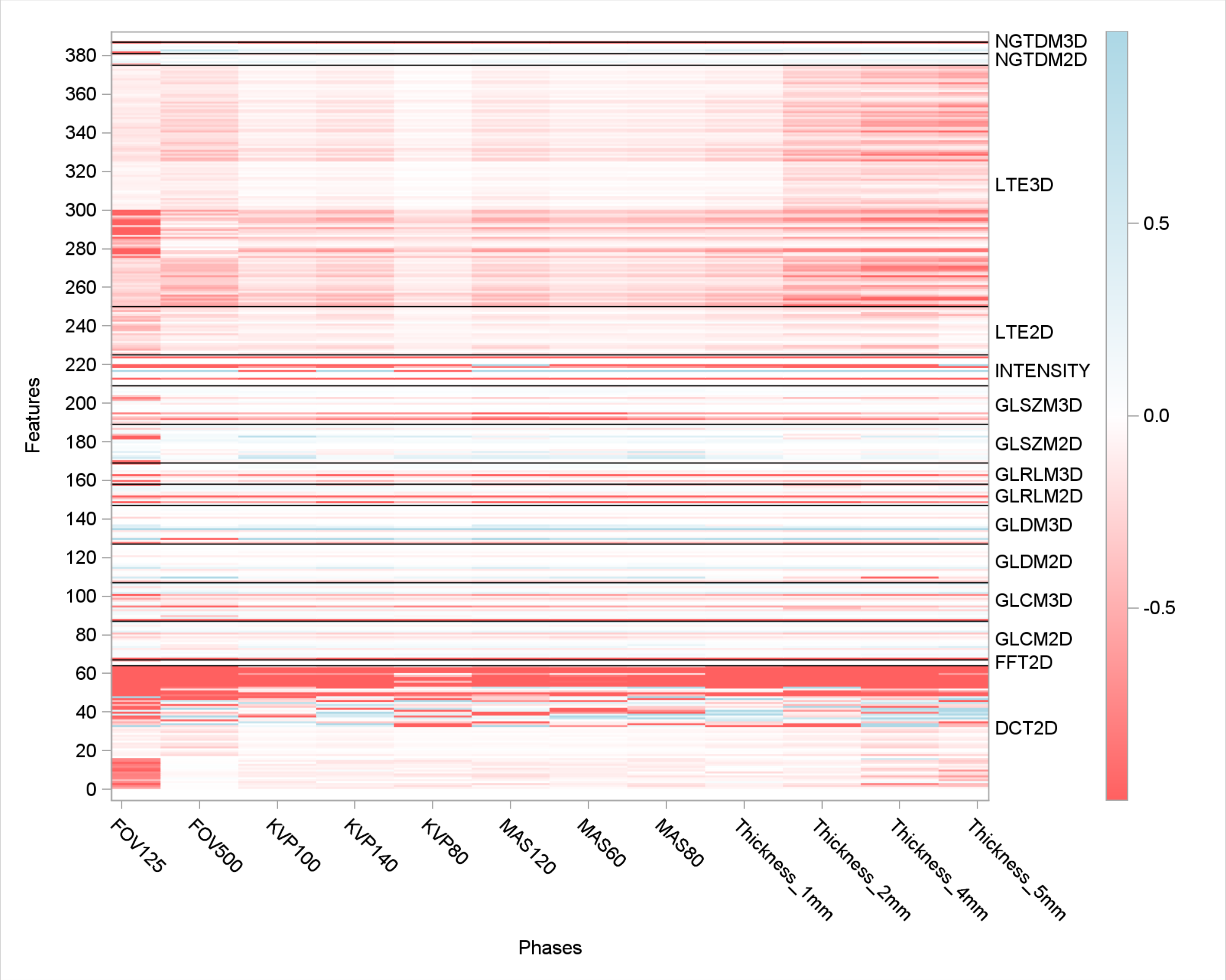

***Figure 8**: Heatmap showing the beta (slope) value of each of the radiomic metrics within the **GE 16 Lightspeed** scanner. Results of the study are presented a heatmap with values ranging from -1 (red) to 1 (blue) i.e., negative linear correlation to positive linear correlation. The texture panel comprised of 387 features belonging to 15 subgroups of texture extraction methods (e.g. GLCM), shown on the y-axis. 12 unique image settings were tested (e.g. FOV125), shown on the x-axis.*

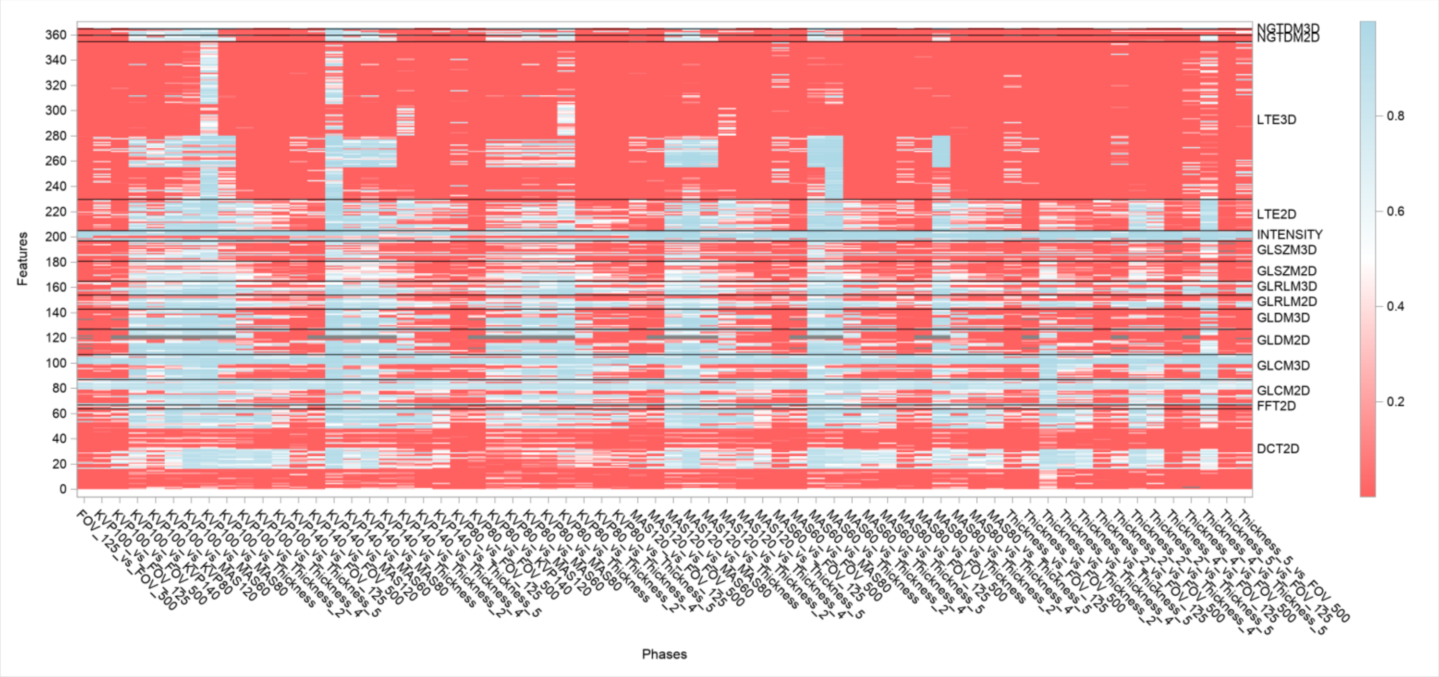

Figure 9: *Heatmap of radiomic metrics robustness, showing the lower-limit of the interclass correlation 2-way mixed with absolute agreement (ICC3.1) of each of the radiomic metrics, within the* ***Siemens Sensation 10*** *scanner. We have calculated the 95% confidence interval for each ICC value. Therefore, if an ICC value from a different feature is below the lower limit of 95% CI, we can claim these two features have different ICC and the feature with lower ICC is statistically lower than the feature with higher ICC. We can also use the 95% CI for each feature to compare with the critical value. Results of the study are presented as a heatmap with values ranging from 0 (red) to 1 (blue) showing the features significantly higher than each of the critical value e.g. 0.9, 0.8, 0.7 etc. The texture panel comprised of 365 features belonging to 15 subgroups of texture extraction methods (e.g. GLCM), shown on the y-axis. The heatmap shows 12 unique image settings (e.g. FOV125), for comparisons across scanners.*

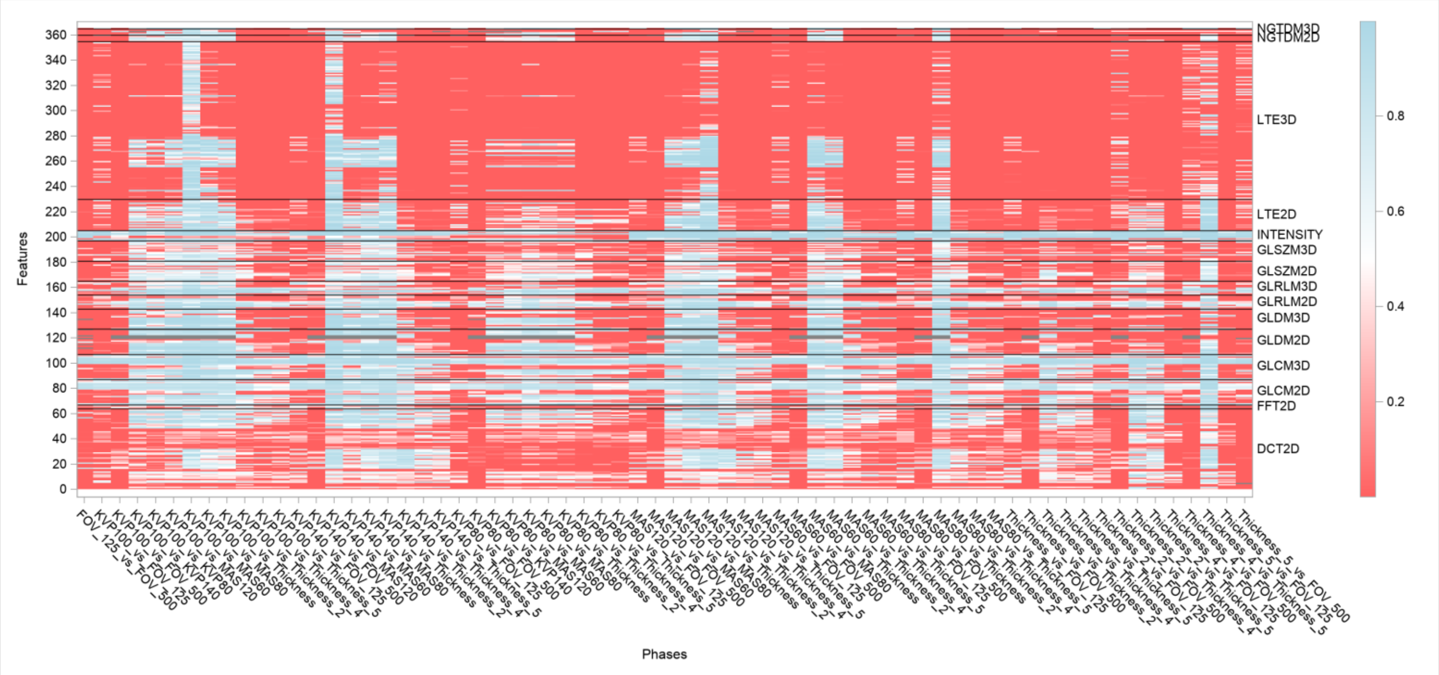

Figure 10: *Heatmap of radiomic metrics robustness, showing the lower-limit of the interclass correlation 2-way mixed with absolute agreement (ICC3.1) of each of the radiomic metrics, within the* ***Philips Brilliance 64*** *scanner. We have calculated the 95% confidence interval for each ICC value. Therefore, if an ICC value from a different feature is below the lower limit of 95% CI, we can claim these two features have different ICC and the feature with lower ICC is statistically lower than the feature with higher ICC. We can also use the 95% CI for each feature to compare with the critical value. Results of the study are presented as a heatmap with values ranging from 0 (red) to 1 (blue) showing the features significantly higher than each of the critical value e.g. 0.9, 0.8, 0.7 etc. The texture panel comprised of 365 features belonging to 15 subgroups of texture extraction methods (e.g. GLCM), shown on the y-axis. The heatmap shows 12 unique image settings (e.g. FOV125), for comparisons across scanners.*

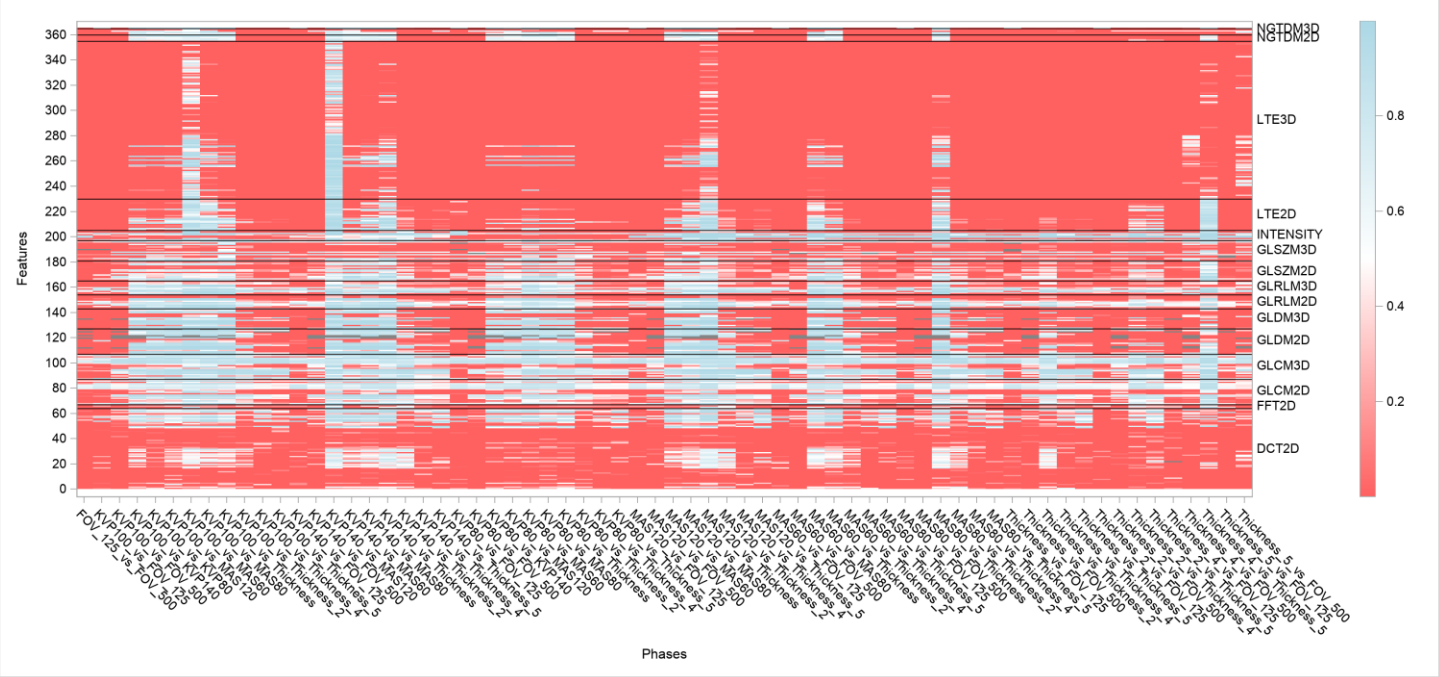

Figure 11: *Heatmap of radiomic metrics robustness, showing the lower-limit of the interclass correlation 2-way mixed with absolute agreement (ICC3.1) of each of the radiomic metrics, within the* **Canon Aquilion Prime 160** *scanner. We have calculated the 95% confidence interval for each ICC value. Therefore, if an ICC value from a different feature is below the lower limit of 95% CI, we can claim these two features have different ICC and the feature with lower ICC is statistically lower than the feature with higher ICC. We can also use the 95% CI for each feature to compare with the critical value. Results of the study are presented as a heatmap with values ranging from 0 (red) to 1 (blue) showing the features significantly higher than each of the critical value e.g. 0.9, 0.8, 0.7 etc. The texture panel comprised of 365 features belonging to 15 subgroups of texture extraction methods (e.g. GLCM), shown on the y-axis. The heatmap shows 12 unique image settings (e.g. FOV125), for comparisons across scanners.*

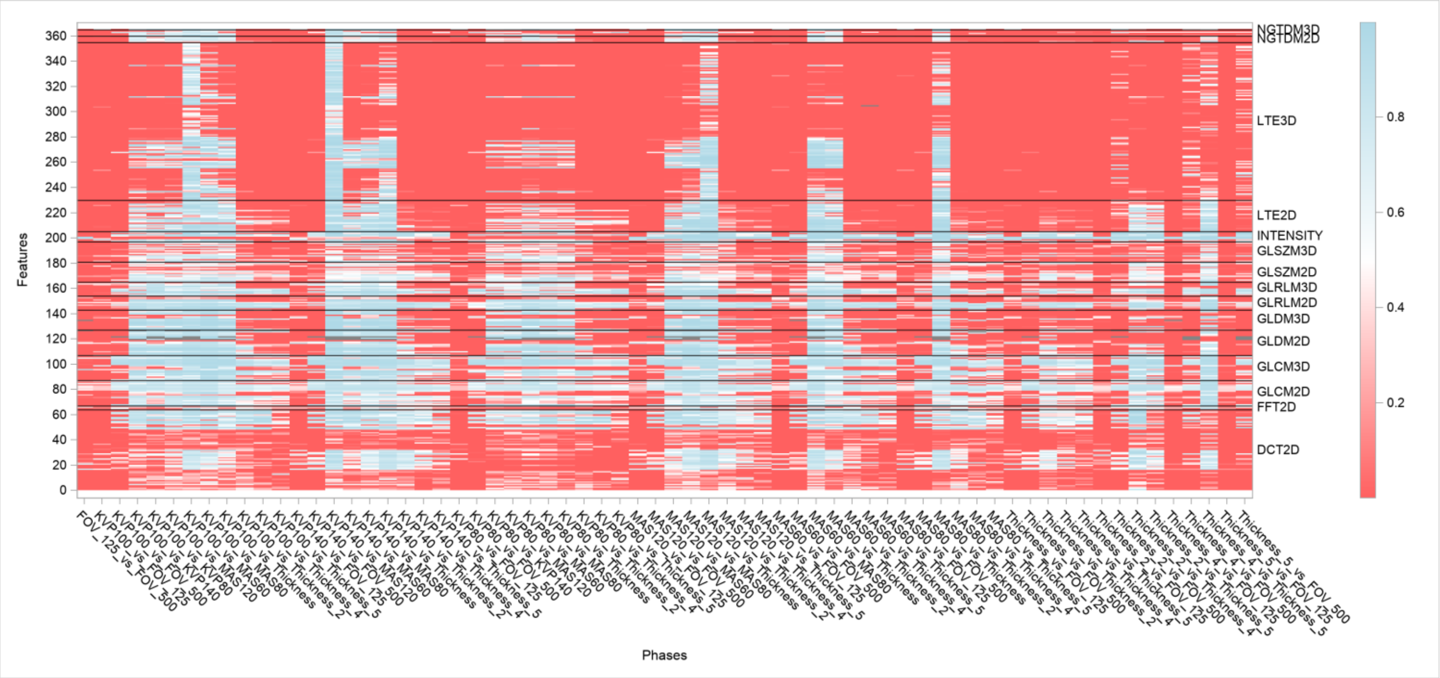

Figure 12: *Heatmap of radiomic metrics robustness, showing the lower-limit of the interclass correlation 2-way mixed with absolute agreement (ICC3.1) of each of the radiomic metrics, within the* ***GE 16 Lightspeed*** *scanner. We have calculated the 95% confidence interval for each ICC value. Therefore, if an ICC value from a different feature is below the lower limit of 95% CI, we can claim these two features have different ICC and the feature with lower ICC is statistically lower than the feature with higher ICC. We can also use the 95% CI for each feature to compare with the critical value. Results of the study are presented as a heatmap with values ranging from 0 (red) to 1 (blue) showing the features significantly higher than each of the critical value e.g. 0.9, 0.8, 0.7 etc. The texture panel comprised of 365 features belonging to 15 subgroups of texture extraction methods (e.g. GLCM), shown on the y-axis. The heatmap shows 12 unique image settings (e.g. FOV125), for comparisons across scanners.*
